# T-bet+ CXCR3+ B cells drive hyperreactive B-T cell interactions in multiple sclerosis

**DOI:** 10.1016/j.xcrm.2025.102027

**Published:** 2025-03-18

**Authors:** Ivan Jelcic, Reza Naghavian, Imran Fanaswala, Will Macnair, Cinzia Esposito, Daniela Calini, Yanan Han, Zoe Marti, Catarina Raposo, Jacobo Sarabia del Castillo, Pietro Oldrati, Daniel Erny, Veronika Kana, Galina Zheleznyakova, Faiez Al Nimer, Björn Tackenberg, Ina Reichen, Mohsen Khademi, Fredrik Piehl, Mark D. Robinson, Ilijas Jelcic, Mireia Sospedra, Lucas Pelkmans, Dheeraj Malhotra, Richard Reynolds, Maja Jagodic, Roland Martin

**Affiliations:** 1 Neuroimmunology and MS Research Section (NIMS), Neurology Clinic, University of Zurich, University Hospital Zurich, 8091 Zurich, Switzerland; 2 Roche Pharma Research and Early Development (pRED), Roche Innovation Center Basel, F. Hoffmann-La Roche Ltd, Basel, Switzerland; 3 SIB Swiss Institute of Bioinformatics, University of Zurich, Zurich, Switzerland; 4 Department of Molecular Life Sciences, University of Zurich, Zurich, Switzerland; 5 Department of Clinical Neuroscience, Karolinska Institutet, Center for Molecular Medicine, Karolinska University Hospital, Stockholm, Sweden; 6 Institute of Neuropathology, University of Freiburg, Freiburg, Germany; 7 Product Development Medical Affairs, Neuroscience and Rare Disease, F. Hoffmann-La Roche Ltd, Basel, Switzerland; 8 Department of Brain Sciences, Imperial College, London, UK; 9 Institute of Experimental Immunology, University of Zurich, Zurich, Switzerland; 10 Therapeutic Design Unit, Center for Molecular Medicine, Department of Clinical Neurosciences, Karolinska Institutet, Stockholm, Sweden; 11 Cellerys AG, Schlieren, Switzerland

**Keywords:** multiple sclerosis, IFN-gamma, T cells, autoreactivity, T-bet+ B cells, CXCR3, hyperreactive B-T cell interaction, B-T cell-enriched clusters, BTEC, self-peptides, autoproliferation, meninges

## Abstract

Multiple sclerosis (MS) is an autoimmune disease of the central nervous system (CNS). Self-peptide-dependent autoproliferation (AP) of B and T cells is a key mechanism in MS. Here, we show that pro-inflammatory B-T cell-enriched cell clusters (BTECs) form during AP and mirror features of a germinal center reaction. T-bet+CXCR3+ B cells are the main cell subset amplifying and sustaining their counterpart Th1 cells via interferon (IFN)-γ and are present in highly inflamed meningeal tissue. The underlying B cell activation signature is reflected by epigenetic modifications and receptor-ligand interactions with self-reactive T cells. AP+ CXCR3+ B cells show marked clonal evolution from memory to somatically hypermutated plasmablasts and upregulation of IFN-γ-related genes. Our data underscore a key role of T-bet+CXCR3+ B cells in the pathogenesis of MS in both the peripheral immune system and the CNS compartment, and thus they appear to be involved in both early relapsing-remitting disease and the chronic stage.

## Highlights

• Autoproliferation (AP) leads to germinal center-like B-T cell clusters in multiple sclerosis
• T-bet+CXCR3+ B cells are strongly enriched in AP and develop into plasmablasts
• AP+ CXCR3+ B cells potentiate Th1 responses and form an inflammatory circuit
• They are associated with CSF cell counts and spinal cord lesions and found in inflamed meninges

## Graphical abstract

**Figure:**
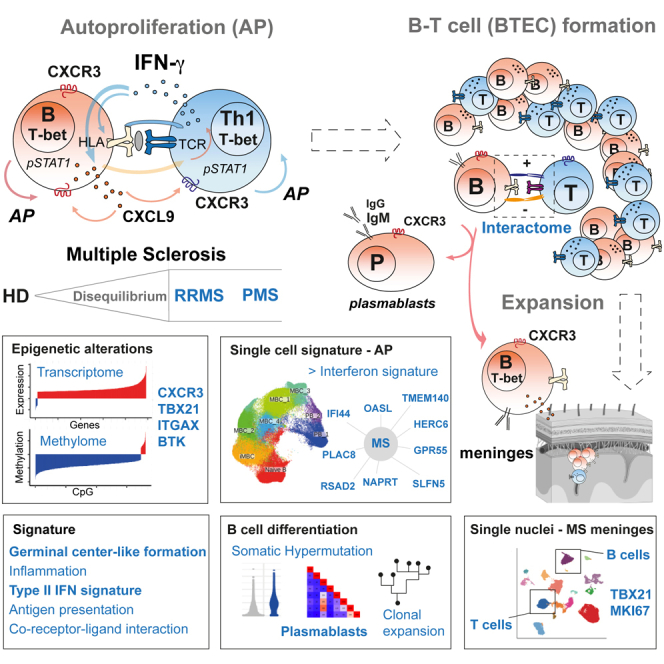

## Teaser

Memory B cells activate brain-homing autoreactive T cells in multiple sclerosis. Here, Jelcic et al. demonstrate that self-reactive B and T cells interact within inflammatory B-T cell clusters. We identify T-bet+CXCR3+ B cells to be the main driver in this inflammatory circuit through IFN-γ in both the periphery and inflamed meninges.

## Introduction

Multiple sclerosis (MS) is an autoimmune disease of the CNS, in which pro-inflammatory autoreactive CD4^+^ T cells are considered major effector cells in orchestrating damaging immune responses.^1^^,^^2^ We recently demonstrated that they engage in tight interactions with memory B cells resulting in pro-inflammatory differentiation, antigen-presenting function, and activation of a brain-homing autoreactive T cell repertoire.^3^ The B and T cell interplay involves the major MS genetic risk factor human leukocyte antigen (HLA)-DR15 and HLA-DR-derived self-peptides from both DR15 allomorphs.^3^^,^^4^ By as yet poorly defined mechanisms, these interactions lead to increased peripheral autoproliferation (AP) and may also be involved in meningeal ectopic lymphoid structures (ELS) in MS.^3^^,^^4^^,^^5^ Foreign antigens from environmental risk factors of MS such as Epstein-Barr virus (EBV) and *Akkermansia muciniphila* can trigger these autoreactive T cells.^4^^,^^6^^,^^7^ Cross-reactivity with the CNS autoantigen RAS guanyl-releasing protein 2 (RASGRP2), expressed by B cells and neuronal cells,^3^^,^^4^ may contribute to peripheral maintenance of autoreactive T cells, migration to the CNS compartment, and target tissue damage. Both the persistence of EBV in memory B cells and evidence from a longitudinal study that EBV infection precedes the development of MS further support this model.^6^^,^^8^

The pathogenic relevance of B cells in MS has been discovered following the remarkable efficacy of B cell depletion in MS patients.^9^^,^^10^ B cells may contribute to MS pathogenesis by inducing peripheral pro-inflammatory immune responses, disrupting peripheral tolerance, driving the invasion of pathogenic B and T cell clones into the CNS, and establishing chronic immune activation in the CNS.^2^^,^^3^^,^^11^^,^^12^^,^^13^^,^^14^ Treatment with the highly effective anti-VLA4 antibody natalizumab, which prevents migration through the blood-brain barrier,^15^ traps autoreactive T cells in the periphery, increases circulating memory B cells,^16^ and not only augments peripheral AP but also frequently leads to clinical rebound activity after discontinuation.^3^^,^^17^

B cells home to perivascular areas of the brain parenchyma and leptomeninges, where they initiate a compartmentalized inflammatory process within the subarachnoid space.^5^^,^^18^ The formation of B-T cell aggregates with features of ELS in the meninges is considered important for sustaining CNS inflammation and disease progression. Forty percent of secondary progressive MS (PMS) autopsy cases show these structures, which likely contribute to the chronic pathology in the underlying cortical gray matter.^5^^,^^19^^,^^20^^,^^21^ Damage can be caused via inflammatory mediators, which diffuse from the subarachnoid space into cortical layers and also via microglial activation and astrocyte loss.^18^^,^^20^ Yet, a more detailed understanding of how ELSs are established, in particular with regard to B-T cell interactions, is still lacking. Recent studies in blood, cerebrospinal fluid (CSF), and meningeal tissue of MS patients point toward an accumulation of T-bet+CXCR3+ B cells.^22^^,^^23^^,^^24^ This B cell subset accumulates in the dura during aging in mice and has been implicated in various autoimmune diseases via altered B cell signaling, potentiated antigen presentation, and increased pro-inflammatory and/or migratory potential.^25^^,^^26^^,^^27^ However, their functional properties and involvement in MS pathogenesis are not fully understood yet due to limitations regarding translation from rodent experimental autoimmune encephalomyelitis (EAE) models to humans, lack of suitable human *in vitro* models, and limited investigations of B cells in CNS tissue.

Here, we present an in-depth characterization and clinical implications of T-bet+CXCR3+ B cells in MS. Due to co-expression of CXCR3 and T-bet in AP+ B cells, we will refer to them as CXCR3+ B cells. B-T interactions induce CXCR3+ B cells via interferon (IFN)-γ with subsequent inflammatory cascade that enhances Th1 effector functions of autoreactive CD4^+^ T cells, the formation of B-T cell-enriched clusters (BTECs), and development of plasmablasts in MS. Finally, we demonstrate the presence of T-bet+CXCR3+ B cells within inflamed meningeal tissue of MS patients, indicating their key role not only during AP but also in compartmentalized CNS inflammation.

## Results

### Autoproliferating cells form BTECs

**Figure 1:**
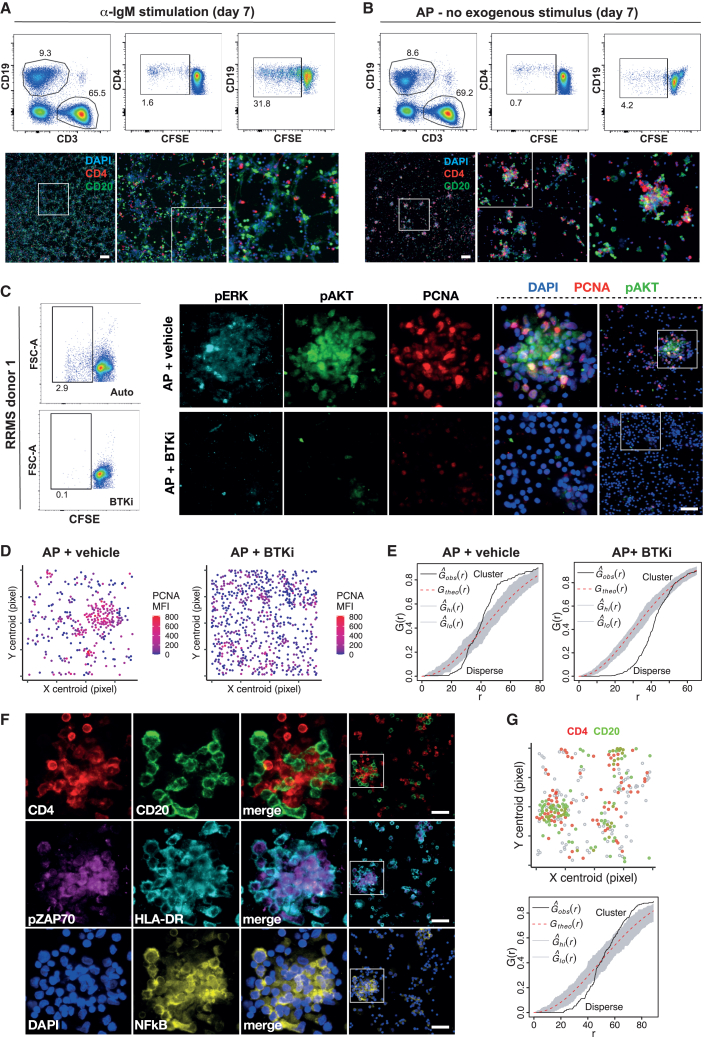
AP results in BTEC formation, which is BTK dependent (A and B) Staining of B cells (CD19 and CD20) and T helper cells (CD3 and CD4) after 7 days of (A) anti-IgM stimulation or (B) AP in PBMCs (natalizumab-treated RRMS = NAT) by flow cytometry (top panel) and by 4i (bottom panel). Nuclei were labeled with DAPI (blue). Images show increasing magnification from left to right, 49-(well), 4-, and 1-site view. CFSE stands for carboxy fluorescein succinimidyl ester. Scale bar: 200 μm. (C) AP response upon vehicle (top) or BTK inhibitor (BTKi) treatment (bottom) assessed by flow cytometry (left) and 4i (right) including staining for pERK (cyan), pAKT (green), and PCNA (red). Representative image from one donor (NAT, *n* = 3). Scale bar: 50 μm. (D) 2D projection of local morphology centroids, colored by the mean fluorescence intensity (MFI) values of PCNA, without (AP) or with BTKi treatment to visualize the spatial intensity distribution of cell proliferation. (E) Computation of nearest neighbor statistics using G(r) function on local morphology centroids, for AP upon vehicle or BTKi treatment to measure deviations from complete spatial randomness in favor of clustering or regular patterns. G(r) values above or below the simulation envelope imply clustering or dispersion, respectively. (F) Representative 4i image of BTECs following AP (*n* = 3 NAT). Images on the right show magnified 1-site view. Scale bar: 50 μm. (G) Top: 2D projection of cell morphology centroids, colored by B cells (green), T cells (red), and unclassified (gray). Bottom: G(r) function computed on cell morphology centroids based on (F). See also Figure S1 and Table S1.

Previously, we have shown increased AP of peripheral blood CD4^+^ memory Th1 cells in MS patients after 7 days of unstimulated *in vitro* culture and that AP involves the HLA-DR15 haplotype and HLA-self-peptide complexes presented on memory B cells.^3^^,^^4^ Apart from T cells, B cells also participate in AP and may play a central role during B-T cell interactions (Figure S1A).^3^ To examine these interactions during AP further, we employed a high content imaging platform, named 4i.^28^ 4i allows high-resolution multiplexed imaging of 2D and 3D cell cultures through repetitive staining cycles and antibody elution (Figures 1 and S1). The 4i protocol follows an embedding procedure, which ensures consistent immobilization of non-adherent lymphocytes throughout multiple cycles (Figures S1B and S1C). Surprisingly, we found significant differences when comparing B cell stimulation by anti-immunoglobulin (Ig)M versus that during AP and in the absence of exogenous stimuli. Although AP and anti-IgM stimulation both resulted in increased T cell activation and cytokine responses in MS,^3^ AP led to BTECs with direct B-T cell contacts (Figures 1A and 1B), while a rather uniform cell distribution without B and T cell engagement resulted from anti-IgM stimulation. BTECs are formed by AP+ cells based on markers of activation/proliferation such as phospho-AK strain transforming (pAKT), phospho-mitogen activated protein kinase 1 (pERK) and proliferating cell nuclear antigen (PCNA) (Figure 1C). Interestingly, inhibition of a key component in B cell function, Bruton’s tyrosine kinase (BTK), via ibrutinib not only abrogated AP^3^ but also prevented BTEC formation (Figure 1C). Computational neighborhood analysis of the spatial cell distribution supports that AP+ cells are indeed crowding locally with enriched PCNA signals within BTECs, whereas the BTK inhibitor ibrutinib blocked this process leading to a sparse distribution of the cells (Figures 1D and 1E). BTECs did not show a clearly distinguishable organization of B and T cells. The strong expression of HLA-DR and phosphorylation of the T cell receptor (TCR)-associated signaling molecule ZAP-70, within the core of BTECs, indicate T cell recognition of self-peptides presented on B cells. The pro-inflammatory nature of BTECs was further demonstrated by the enhanced nuclear factor κB (NF-κB) expression and partial translocation into the nucleus (Figures 1F, 1G, and S1D–S1G).

### AP+ B cells are increased in MS and show germinal center-like characteristics

**Figure 2:**
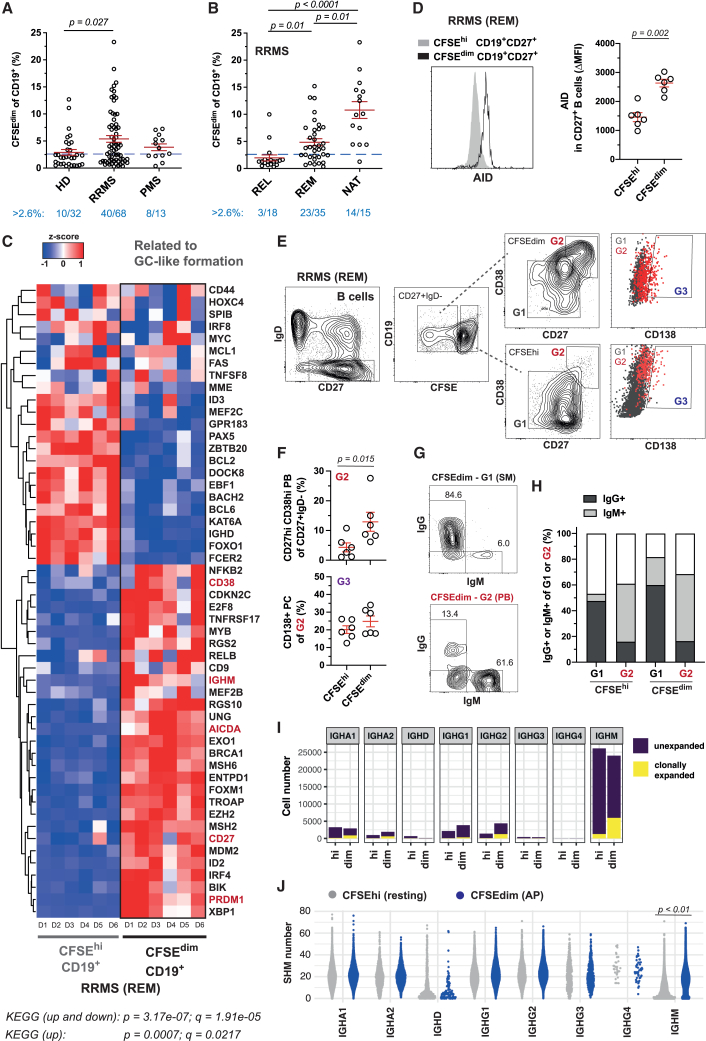
AP+ B cells are increased in MS and bear characteristic features of germinal center-like formation (A and B) (A) Frequency of AP+ B cells in HD (*n* = 32), RRMS (*n* = 68, REM/REL nihil and NAT), and PMS patients (*n* = 13, nihil) (mean ± SEM; Kruskal-Wallis test). (B) Frequency of AP+ B cells in RRMS group, untreated RRMS in relapse (REL, *n* = 18) or remission (REM, *n* = 35), and natalizumab-treated RRMS (NAT, *n* = 15) (mean ± SEM; Kruskal-Wallis test). Dotted line indicates mean value of AP+ B cells and below the number of donors with >2.6% mean value. (C) RNA-seq of CFSE^hi^ and CFSE^dim^ (AP+) B cells (*n* = 6 REM) for a predefined gene set characteristic for germinal center (GC) formation. The differential expression is expressed by the *Z* score based on the reads per kilobase of transcript per million reads mapped (RPKM) values. The gene set was tested as customized KEGG pathway for significance (Fisher’s exact test). (D) Intracellular AID expression in CFSE^hi^ and CFSE^dim^ (AP+) memory B cells. (Left) Representative histogram plot; (right) MFI of AID expression corrected by isotype control (*n* = 6 REM; mean ± SEM; Mann-Whitney *U* test). (E) Gating strategy of switched memory B cell (G1), plasmablasts (G2), and plasma cells (G3) in CFSE^hi^ and CFSE^dim^ B cells upon AP. (F) Frequency of plasmablasts/-cells following AP (*n* = 6 REM; mean ± SEM; Mann-Whitney *U* test). (G and H) Expression of IgG and IgM upon AP. (G) Representative histogram plot from one donor and (H) distribution across B cell subsets and donors (*n* = 5 REM). (I) scVDJ-sequencing-derived BCR isotype usage in AP+ and resting B cells across donors (*n* = 16 donors; HD: *n* = 5, REM: *n* = 6, NAT: *n* = 5) and the proportion of clonally expanded B cells. (J) SHM counts in AP+ and resting B cells stratified by their BCR isotype (*n* = 16) (t test with false discovery rate [FDR] < 0.01 and a location shift of 1 base). See also Figures S2 and S6 and Table S1.

We observed an increase of AP+ B cells during relapsing-remitting MS (RRMS) compared to healthy donors (HD) (Figure 2A) as shown previously for AP+ T cells.^3^ In line with the T cell data,^3^ AP+ B cells were significantly lower in untreated RRMS patients during relapse (REL), that is, during new brain inflammation. In contrast, their frequency increased in untreated RRMS patients during remission (REM) and even more in RRMS patients treated with natalizumab (NAT) (Figure 2B and Table S1). Moreover, we also found increased AP+ B cells in PMS patients, in whom disability accrual occurs independent of relapses, and inflammation is likely compartmentalized in the CNS. In fact, the frequency of donors having >2.6% (median response) AP+ B cells was comparable in both MS groups (59% RRMS and 62% PMS vs. 31% HD). However, in progressive MS (PMS; secondary- (SPMS) or primary progressive (PPMS) MS), less donors show strongly increased AP (Figure 2A).

Interestingly, differentially expressed genes (DEGs) in AP+ versus resting B cells from RRMS (REM) patients were enriched for genes involved in germinal center (GC) formation, which usually takes place in secondary lymphoid organs (SLOs). DEGs included those essential for immunoglobulin class switch recombination and somatic hypermutation (SHM) (*AICDA*, *EXO1*, *UNG*, *MSH2*, and *IRF4)*, NF-κB signaling (*RELB* and *NFKB2*), and B cell maturation (*CD27*, *CD38*, *PRDM1*, and *XBP1*) (Figure 2C). The master regulator for GC formation, AID (*AICDA*), was also highly abundant at the protein level in AP+ B cells (Figure 2D). Noticeably, AP+ B cells also contained a large proportion of CD27^hi^CD38^hi^ plasmablasts and even CD138+ plasma cells (Figures 2E and 2F). Switched memory B cells were mainly IgG positive, and plasmablasts were mainly IgM positive (Figures 2G and 2H). Single-cell variable diversity joining (VDJ) segment sequencing of AP+ B cells identified *IGHM* as the most abundant isotype, and, together with *IGHA2*, *IGHG1*, and *IGHG2*, it was more prevalent in AP+ B cells (Figure 2I). Clonal expansions were more pronounced in *IGHA1*-, *IGHA2*-, *IGHG2*-, and *IGHM*-expressing AP+ B cells (Figure 2I), but SHMs were only increased in *IGHM* of AP+ cells, indicating preferential stimulation of IgM+ B cells during BTEC formation and B cell maturation during AP (Figure 2J).

### Patients with high AP+ B cells show elevated CSF lymphocyte counts and spinal cord lesions

Next, we wondered if increased AP+ B cells are related to clinical findings. We examined patients in remission divided by the median of the AP response (>3.6% versus <3.6% AP+ B cells). Basic CSF parameters of 24 MS patients within 2 years before collection of peripheral blood for testing AP identified more patients and a 10-fold higher likelihood of pathologically elevated CSF cell count (>4 cells/μL) in those with increased AP (odds ratio 10.0, 95% confidence interval 1.4%–69.3%, *p = 0.020*) and also significantly higher mean CSF cell numbers (12.9 ± 20.4 cells/μL) compared to patients with low AP+ B cells (4.5 ± 2.6 cells/μL, *p = 0.032*) (Figures S2A–S2C). No other basic clinical CSF parameter including intrathecal IgG, IgA, or IgM production, CSF/serum albumin ratio, or total CSF protein differed between these groups (Figures S2D–S2T). Clinical and radiological parameters in 34 MS patients at collection of peripheral blood for testing AP and last follow-up (approximately 4 years later) showed significantly more spinal cord lesions in patients with increased AP+ B cells. No other demographic, clinical, and/or other radiological parameter differed between these two groups (Table S2).

### AP leads to an IFN-γ-driven T-bet+CXCR3+ B cell phenotype

**Figure 3:**
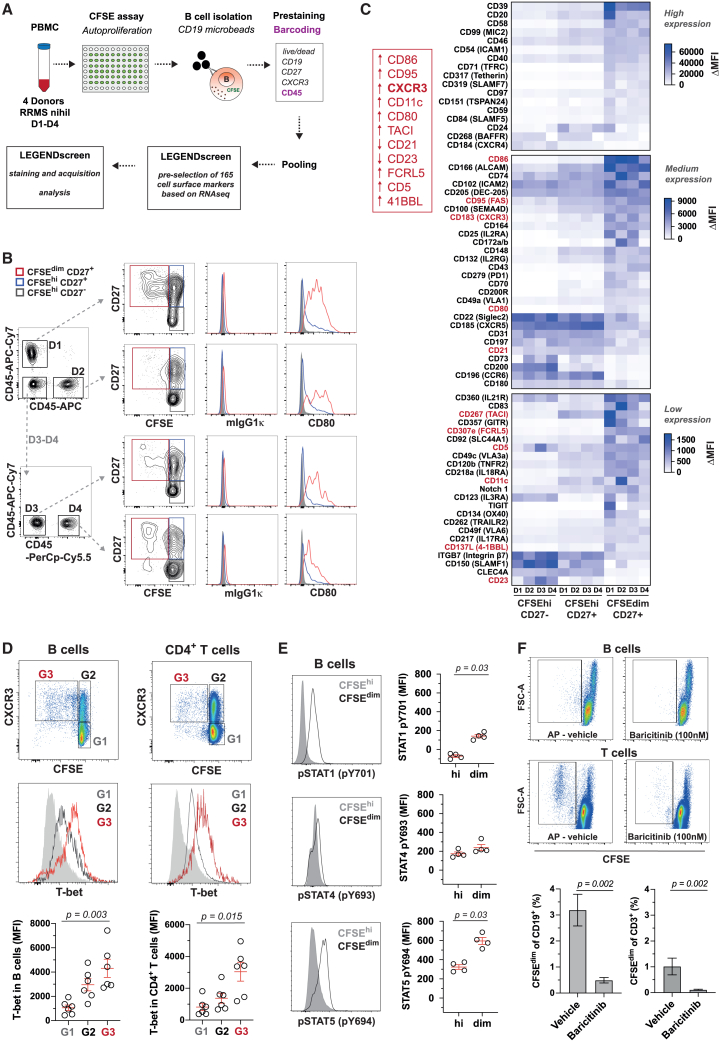
AP+ B cells reveal abundant expression of IFN-γ-driven markers and dependency on JAK-STAT pathway (A) Workflow of isolated, pre-stained, and sample barcoded CFSE-labeled B cells following AP (*n* = 4 REM) for surface marker screening using LEGENDscreen. (B) Gating strategy for sample deconvolution based on CD45 barcoding, subgating on CD27^−^CFSE^hi^ naive resting B cells (gray), CD27+CFSE^hi^ memory resting B cells (blue), and CD27+CFSE^dim^ memory AP+ B cells (red). Histogram plots of CD80 expression and respective isotype control. (C) Heatmap summarizing surface marker expression levels in B cell subsets of all 4 donors (D1–D4). Categorization in low, intermediate, and high expressing proteins for better visualization. The target protein median fluorescence intensity (MFI) was corrected with its respective isotype control. Only surface markers with MFI > 250 and MFI ratio changes of >1.5 or <0.5 are shown. The red box highlights characteristic protein expression of age-associated B cells. (D) Intracellular T-bet expression in CXCR3-CFSE^hi^ (G1), CXCR3+CFSE^hi^ (G2), and CXCR3+CFSE^dim^ (AP+) (G3) B cells and T helper cells. (Upper) Representative plots from one patient and (bottom) MFI of each donor (*n* = 6 REM; mean ± SEM; Kruskal-Wallis test). (E) Intracellular phosphorylation of STAT1, STAT4, and STAT5 in CFSE^hi^ and CFSE^dim^ B cells upon AP. (Left) Representative histogram plot and (right) MFI in all donors (*n* = 4 REM; mean ± SEM; Mann-Whitney *U* test). (F) AP of B and T cells upon treatment with vehicle control or JAK-STAT inhibitor baricitinib (100 nM). (Upper) Representative dot plots from one patient and (bottom) summary of all donors (*n* = 6 REM; mean ± SEM; Kruskal-Wallis test). See also Figure S3 and Table S1.

Next, we characterized the phenotype of AP+ B cells in MS by detailed flow cytometry-based analysis using backbone markers (CD19 and CD27) and 165 pre-selected surface markers (LEGENDscreen, selected based on transcriptional B cell expression) in a pool of four CD45-barcoded RRMS (REM) patient samples (Figure 3A). We identified differentially expressed surface receptors of AP+ B cells versus resting memory and naive B cells (Figures 3B and 3C). Among the upregulated proteins in AP+ B cells were receptors involved in cell-cell interaction, such as the highly expressed CD58, ICAM1, SLAMF7, and CD84; intermediate expressed CD86, CD279 (PD-1), CD70, and CD80; lowly expressed CD357 (GITR), TIGIT, CD134 (OX-40), and CD137L (4-1BBL), or cell adhesion proteins such as CD166 (ALCAM), CD164, and CD49c (VLA3a) (Figure 3C). Of note, CD20 expression was increased in AP+ B cells indicating that they might be particularly sensitive to anti-CD20 depletion therapy. Interestingly, we observed a set of upregulated proteins in AP+ B cells, namely CXCR3, CD307e (FCRL5), CD95, CD86, partially CD11c, and decreased CD21 and CD23 (Figure 3C), which are considered key phenotypic markers of a B cell subset, known as age-associated B cells (ABCs), extrafollicular or atypical B cells in chronic infection and autoimmune diseases.^26^^,^^29^^,^^30^ Pre-gating on CXCR3 supported the preferential expression of these markers in AP+ versus resting B cells and also the upregulation of several co-receptor/ligands facilitating T cell interaction (Figures S3A, S3B and S3C). Previously, ABCs have been considered immunosenescent cells following chronic antigenic stimulation and recognized as a distinct subpopulation arising from various stages (CD27^+^ or CD27^−^).^31^ ABCs express the transcription factor T-bet, which we found highly upregulated as well in AP+ CXCR3+ B cells and similarly as in AP+ Th1 cells (Figure 3D). Besides the high T-bet expression in AP+ B cells, phosphorylated STAT1 and STAT5 were also increased (Figure 3E). Phosphorylation of STAT1 is induced by IFN-γ signaling and has been shown to be essential for GC formation,^32^ while STAT5 is induced by γ-chain cytokines such as interleukin (IL)-2 and IL-21 and is critical during cell-cycle progression. Accordingly, our inhibition of the JAK-STAT pathway by baricitinib, a selective JAK1/JAK2 inhibitor targeting IFN-mediated signaling, leads to almost complete abrogation of AP in B and T cells, supporting that IFN-γ signaling is at the core of BTEC formation (Figure 3F).

### AP+ CXCR3+ B cells in MS potentiate the Th1/IFN-γ response

**Figure 4:**
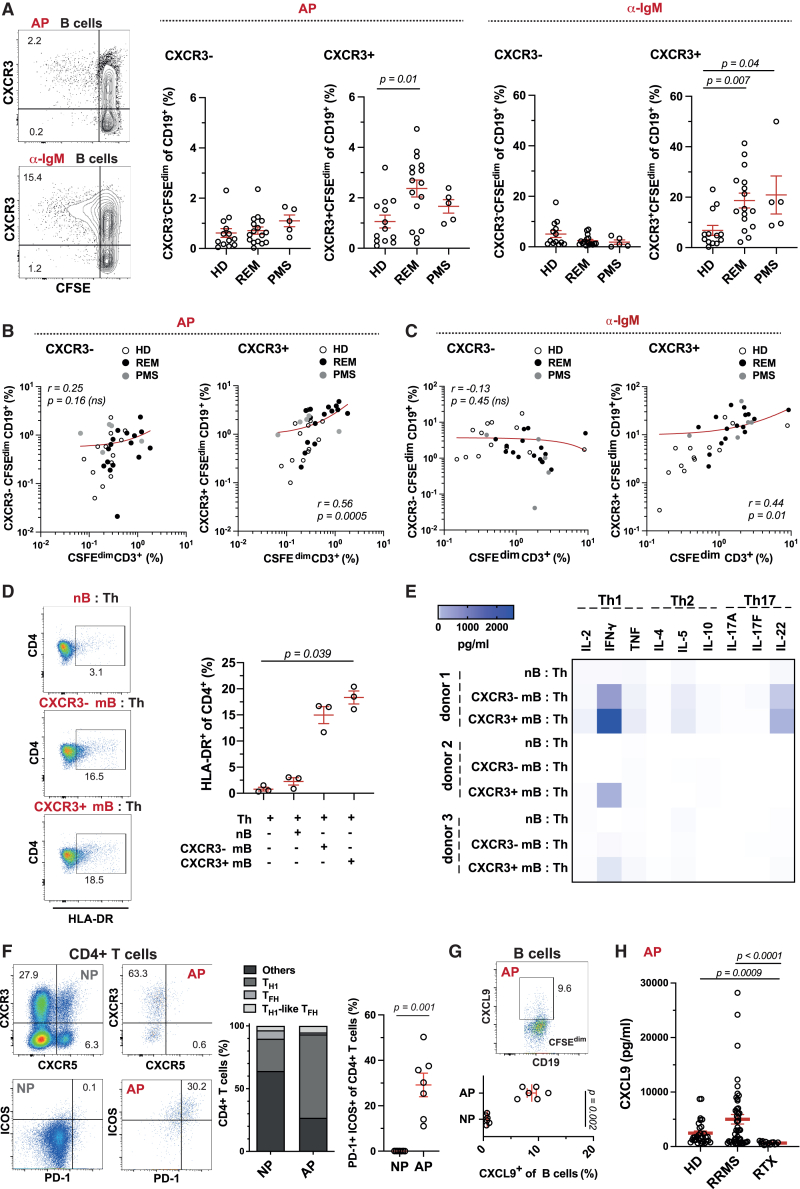
AP+ CXCR3+ B cells are enriched in RRMS and PMS and promote Th1 responses (A) Frequency of CXCR3− or CXCR3+ B cells in HD (*n* = 13), REM (nihil; *n* = 16), and PMS (nihil; *n* = 5) upon AP (left) or IgM stimulation (right) (mean ± SEM; Kruskal-Wallis test). Representative gating strategy is shown from one donor. (B and C) Correlation of CFSE^dim^ B and T cells after (B) AP and (C) anti-IgM in HD (*n* = 13), REM (*n* = 16), and PMS (*n* = 5) (Pearson’s correlation test). (D) Activation of CD4^+^ T cells (HLA-DR+) upon AP co-culture with autologous CD27^−^ naive (nB), CXCR3-CD27^+^, or CXCR3+CD27^+^ memory (mB) B cells (NAT *n* = 3; Kruskal-Wallis test). (E) Secretion of Th1, Th2, and Th17 cytokines after AP of B-T cell co-cultures (NAT *n* = 3). Levels of cytokine secretion are shown as heatmap. (F) Frequency of Th1 and Tfh cells upon AP. (Left) Representative dot plots from one patient and (right) distribution across donors in CFSE^hi^ (non-proliferating = NP) and CFSE^dim^ (AP) CD4^+^ T cell phenotypes (*n* = 7 NAT; mean ± SEM; Mann-Whitney *U* test). (G) Intracellular expression of CXCL9 in CFSE^hi^ (NP) and CFSE^dim^ (AP+) B cells. (Top) Representative dot plot from one donor and (bottom) summary of all donors (REM *n* = 6, mean ± SEM; Mann-Whitney *U* test). (H) Secretion of CXCL9 upon AP in HD (*n* = 30), RRMS (*n* = 47: REM *n* = 32; NAT *n* = 15), and rituximab-treated RRMS (RTX *n* = 14) (mean ± SEM; Kruskal-Wallis test). See also Figure S4 and Table S1.

AP+ CXCR3+ B cells are significantly increased among the AP+ B cells in untreated RRMS (REM) patients compared to HD. This increase was observed after both AP and B cell receptor (BCR) stimulation by anti-IgM (Figure 4A). In contrast, the frequency of AP+ CXCR3− B cells did not differ between these groups. AP+ CXCR3+ B cells correlated also more strongly with AP+ T cells than resting B cells, suggesting close interactions of AP+ T cells with AP+ CXCR3+ B cells (Figures 4B and 4C).

Next, we addressed if CXCR3+ B cells stimulate CD4^+^ T cells more efficiently than their CXCR3− counterpart by co-culturing CXCR3− or CXCR3+ memory B cells with autologous CD4^+^ T cells. T cell activation as measured by HLA-DR upregulation was higher upon co-culture with memory B cells but without significant difference between CXCR3+ and CXCR3− cell subsets (Figure 4D). In contrast, a strong induction of IFN-γ was observed when T cells were cultured with CXCR3+ memory B cells (Figure 4E), indicating that CXCR3+ B cells amplify Th1 responses more efficiently. IFN-γ secretion and CXCR3 expression may be characteristic for various T cell lineages (e.g., classical and non-classical Th1 and T follicular helper [Tfh] cells), and formation of GC-like structures should involve Tfh cells. However, AP+ T cells were predominantly enriched for CXCR3+ Th1 cells and not CXCR5+ Tfh cells. Yet, AP+ Th1 cells expressed high levels of inducible T-cell co-stimulator (ICOS) and programmed death -1 (PD-1) characteristic for Tfh cells (Figure 4F). Among other top IFN-γ-inducible genes upregulated in AP+ B cells was CXCL9 (Figure 4G). The CXCR3 ligand CXCL9 is known to specifically attract CXCR3+ Th1 cells and may be important for BTEC formation and likely also CNS homing of CXCR3+ B and T cells. Consistent with this assumption, B cell depletion by rituximab in MS patients led to a drop in CXCL9 production (Figure 4H) and thus provides an additional explanation why T cell activation and AP are reduced by anti-CD20 treatment.^3^

Transcriptome analyses of AP+ T cells pointed toward enhanced T cell co-stimulation and increased NF-κB signaling that may foster B-T cell engagement and BTEC formation (Figures S4A–S4C). Among the many differentially expressed co-stimulating and co-inhibitory receptor genes in AP+ T cells, the OX-40 and 4-1BB pathways were strongly upregulated (Figures S4B and S4D). Both receptors were expressed on a fraction of AP+ Th cells (Figure S4E). Interestingly, the transcription (Figure S4F) and surface expression (Figure S3) of their ligands, 4-1BBL and OX-40L, were upregulated on AP+ B cells supporting a role of the receptor-ligand pairs OX-40/OX-40L, 4-1BB/4-1BBL, and CXCR3/CXCL9/CXCL10 in BTECs (Figure S4F).

### Upregulated pro-inflammatory genes such as *CXCR3* and *TBX21* show epigenetic modifications in AP+ B cells

**Figure 5:**
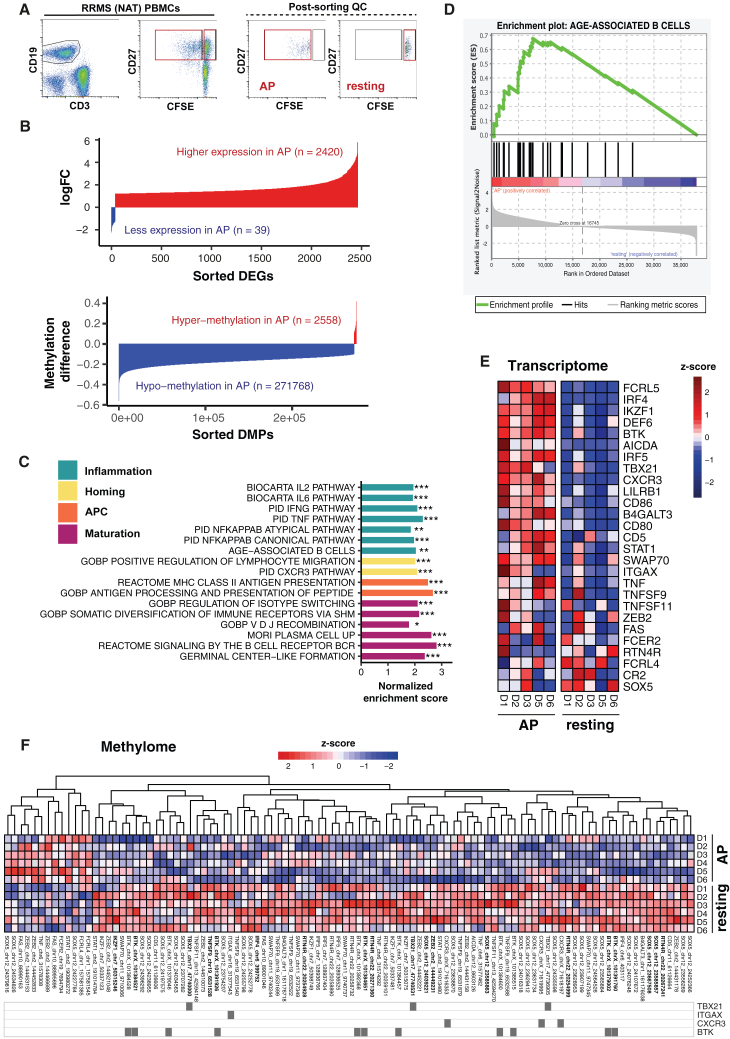
AP+ B cells display transcriptional and epigenetic changes in key inflammatory markers of CXCR3+ B cells (A) Gating strategy and purity from sorting of resting (CFSE^hi^) and AP+ (CFSE^dim^) memory B cells (representative, *n* = 6 NAT). (B) Global transcriptome (*n* = 5) and methylome changes (*n* = 6) in AP+ vs. resting memory B cells. (C) Pathway analysis of DEGs in AP+ B cells. ∗: FDR < 0.01, ∗∗: FDR < 0.001, ∗∗∗: FDR < 0.0001. (D) Gene set enrichment analysis of genes characteristic for age-associated B cells (ABCs). The enrichment score is illustrated in green with differences in expression ranked from high (red) to low (blue). (E) Heatmap illustrating expression of ABC-related genes in AP+ vs. resting memory B cells. The *Z* score is based on DESeq2 normalized gene expression values. (F) Heatmap illustrating methylation levels of CpGs in key ABC genes in AP+ vs. resting memory B cells. All CpGs located within promoter, promoter-flanking, and enhancer regions of genes with *p* < 0.01 are shown; differential methylation positions (DMPs) with *p* value < 0.001 and difference ≥ 10% are highlighted in bold. See also Figures S2 and S4 and Tables S1 and S2.

We examined the relationship of transcriptional and epigenetic changes in sorted AP+ vs. resting memory B cells from NAT patients (Figure 5A) and assessed simultaneously their methylome and transcriptome. We found overall more profound gene expression and hypomethylation in AP+ memory B cells, suggesting a link between epigenetic modifications and transcriptional activity (Figure 5B). Gene set enrichment analysis of DEGs in AP+ memory B cells revealed pathways of inflammatory cytokine responses including IFN-γ and NF-κB signaling; antigen presentation; migration; and B cell maturation including plasmablast development, VDJ recombination, and SHM (Figure 5C). Interestingly, the upregulated DEGs included many key genes of ABCs,^26^ and generally there was an enrichment of those genes in AP+ B cells (Figures 5D and 5E). ABC-related genes were hypomethylated particularly in AP+ cells. We identified 491 CpGs (*p* < 0.01) in gene regions of which 453 were hypo- and 38 were hypermethylated in AP+ B cells. 102/491 CpGs were located in promotor, promoter-flanking, and enhancer regions, including several hypomethylation sites in *CXCR3*, *ITGAX* (CD11c), *IRF5*, *BTK*, and *SOX5* genes and also in *TBX21* (T-bet) and *STAT1*, in AP+ B cells indicating that IFN-γ response genes are also epigenetically modified (Figure 5F). The accumulation of hypomethylated sites in *BTK* and *SOX5* may explain the enhanced BCR signaling in autoimmune conditions and in terminal B cell differentiation, respectively.^33^^,^^34^

### CXCR3+ B cells are enriched for IFN-γ-response genes in MS

**Figure 6:**
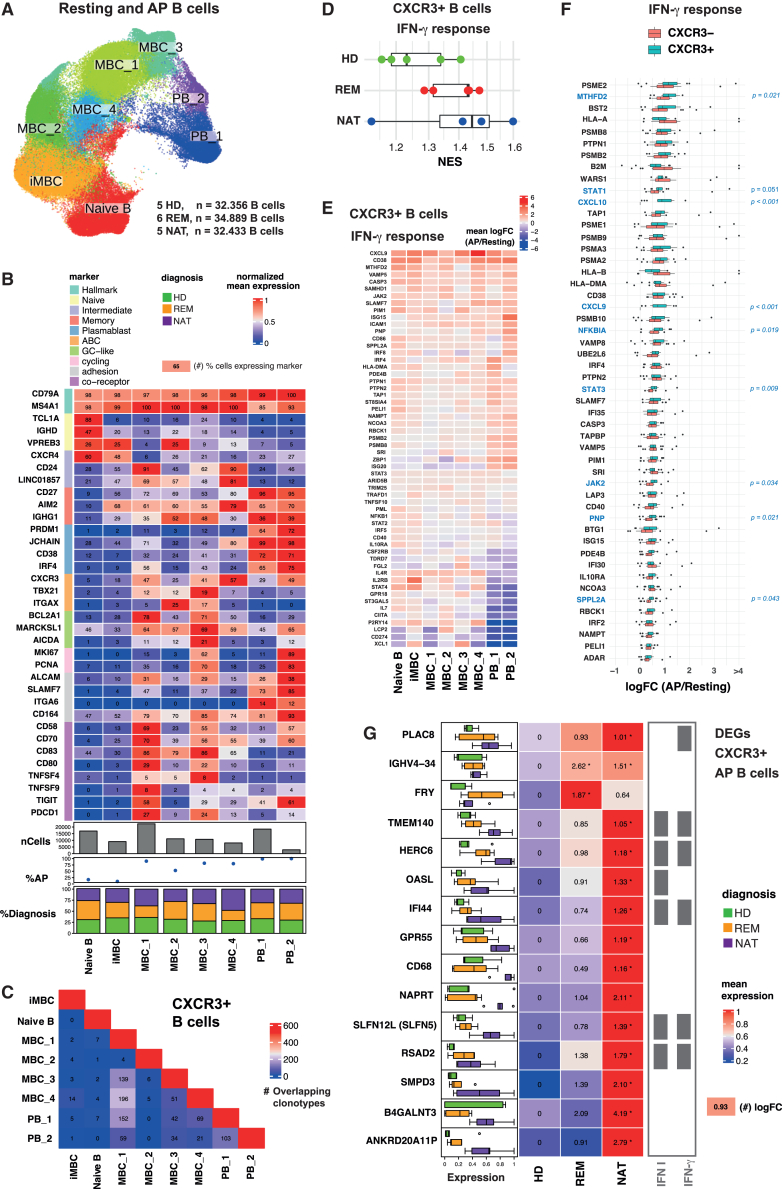
Single-cell RNA-seq of AP+ CXCR3+ B cells reveals abundant enrichment of IFN-γ-response genes in MS (A) Uniform Manifold Approximation and Projection (UMAP) of 99.678 single cells from equal sample size collected from resting and AP+ B cells of HD (*n* = 5), REM (*n* = 6), and NAT (*n* = 5) samples, showing the annotation of 8 clusters: naive B cells, intermediate memory B cells (iMBCs), memory B cells (MBC_1 - MBC_4), and plasmablasts (PB_1 - PB_2). Each dot corresponds to a single cell, colored according to cell cluster. (B) Key phenotypic marker gene expression and abundance across B cell subsets. The color code depicts the normalized mean expression of the gene, and the numbers denote the frequency of cells for which each gene is detected. (C) Clonotypes that span various CXCR3+ B cell subsets. The numbers indicate the number of clonotypes spanning across two respective B cell subsets. (D) IFN-γ response gene set enrichment analysis for CXCR3+ B cells in HD,REM and NAT. (E) DEGs (FDR < 0.01, mean expression < 5% quantile) of the IFN-γ response pathway (Gene ID: 3458) between AP+ and resting CXCR3+ B cells across the different B cell subsets. Color coding depicts fold change of AP vs. resting. (F) Differential gene expression analysis between AP+ and resting within CXCR3− (red) and CXCR3+ (blue) B cells, respectively. Only upregulated genes in AP with logFC > 0.5 are shown with significant DEGs highlighted in blue (Wilcoxon test). (G) DEGs that are exclusively upregulated in AP+ CXCR3+ B cell subsets of MS and not in AP+ CXCR3− B cells. Boxplots describe the pseudobulked expression level and donor-to-donor variability in HD (green), REM (yellow), and NAT (purple). The heatmap shows condition-wise mean expression of the DEGs with logFC (AP vs. resting) values indicated inside the boxes. ∗: FDR < 0.05. See also Figures S5 and S6 and Tables S1, S3, and S4.

In order to dissect the functional and phenotypic aspects of CXCR3+ B cells and their molecular signatures between HD and MS by single-cell RNA and VDJ sequencing (scRNA-seq/scVDJseq) of equal cell numbers of resting and AP+ B cells from HDs, RRMS (REM), and RRMS (NAT) donors, we obtained a total of 99.678 cells post-QC and comparable cell numbers from all three donor groups (Figure 6A). Unsupervised clustering of all B cells resulted in eight major cell clusters (Figure 6A) and annotated them based on selected canonical gene markers as naive-state B cells (*TCL1+ IGHD+*); intermediate memory B cells (iMBCs) (*CXCR4*^*+*^*CD24*^*hi*^ *LINC01857*^*+*^*TCL1A*^*low*^); memory B cells (*AIM2*^*+*^) including MBC_1 (*CD24*^*+*^*co-receptor*^*hi*^), MBC_2 (*VREB3*^*+*^*ITGAX*^*+*^*IGHG1*^*hi*^), MBC_3 (*AICDA*^*+*^*TBX21*^*hi*^*MKI67*^*hi*^*co-receptor*^*hi*^), and MBC_4 (*CXCR3*^*hi*^*CD24*^*hi*^); plasmablasts (*JCHAIN*^*+*^) PB1 (*ITGA6*^*hi*^); and plasmablasts/plasma cells PB2 (*MKI67*^*hi*^*IRF4*^*hi*^*PRDM1*^*hi*^*TIGIT*^*hi*^*adhesion marker*^*hi*^) (Figure 6B). Resting B cells contained predominantly naive, iMBC, and MBC_2 clusters, whereas AP+ B cells were enriched in MBC_1, MBC_3, and MBC_4 clusters and contributed to most of PB_1–2 clusters (Figures S5A and S5B). We did not observe major cell phenotype variations between HDs and MS groups, with the exception of MBC_4, which derived almost half from NAT (Figure 6B). The data-driven signature genes for all B cell subtypes were agnostic regarding cell origin (i.e., resting or AP) and donors (Figures S5C–S5E and Table S3). Regarding ABC markers, all MBC_1–3 subsets showed detectable levels of *TBX21* (T-bet) and MBC_2–3 showed higher levels of *ITGAX* (CD11c), and *CXCR3* was broadly expressed throughout the MBC and PB stages, but the highest in MBC_4 (Figure 6B). CXCR3+ B cells were highly enriched in AP+ B cells and tended to be more abundant in the MS groups (Figures S5F and S5G).

Using BCR repertoire analysis via scVDJseq, we found a strong overlap of shared clonotypes in MBC_1 with MBC_3, MBC_4 and PBs, yet more CXCR3+ MBC_3 clonotypes than CXCR3− that develop preferentially to plasmablasts (Figures 6C and S6A). Expanded AP+ CXCR3+ B cell clonotypes showed differentiation across the various memory B cell stages, isotype switching, and also maturation toward somatically hypermutated *IGHM*-, *IGHA2*-, and *IGHG2*-expressing plasmablasts suggesting their differentiation during AP (Table S4). Among AP+ CXCR3+B cells, *IGHG* usage was more prevalent in MBC_3, *IGHM* in MBC_1 and MBC_4, and *IGHA* in MBC_1 and PB_2 compared to CXCR3− cells (Figures S6B and S6C).

The aforementioned data suggest an important role for IFN-γ in BTEC and CXCR3+ B cell formation, and accordingly gene set enrichment analysis disclosed that the IFN-γ response pathway in CXCR3+ B cells gradually increased in REM and NAT relative to HDs (Figure 6D). IFN-γ-response genes were upregulated across all AP+ B cell subsets, yet plasmablasts showed a different expression pattern (Figure 6E). Among the top upregulated IFN-γ-response genes, AP+ CXCR3+ B cells expressed higher levels of *CXCL9*, *CXCL10*, and numerous other key IFN-γ-response genes (*STAT1*, *STAT3*, *PNP*, *NFKBIA*, and *JAK2*) (Figure 6F). Interestingly, a set of genes was significantly elevated in MS compared to HD and specifically in CXCR3+ B cells. Among these 15 DEGs were 7 IFN-response genes (*TMEM140*, *HERC6*, *OASL*, *IFI44*, *RSAD2*, *PLAC8*, and *SLFN12L* [*SLFN5*]) indicating stronger responsiveness of the CXCR3+ B cell subset to IFN signaling in MS (Figures 6G and S5H). Other DEGs have been shown to promote pro-inflammatory responses and plasma cell maturation (*GRP55*, *NAPRT*, and *SMPD3*).^35^^,^^36^^,^^37^ Of note, we also observed a significant enrichment of the BCR heavy-chain gene IGHV4-34 in both MS groups compared to HD (Figure 6G), which had previously been reported to be overabundant in MS CSF.^38^^,^^39^^,^^40^ Preferentially enriched BCR V gene segments of CXCR3+ B cells of MS patients indicate convergent selection as well (Figure S6D).

### T-bet+ B cells and IFN-γ+ T cells are present in highly inflamed meningeal tissue in MS

**Figure 7:**
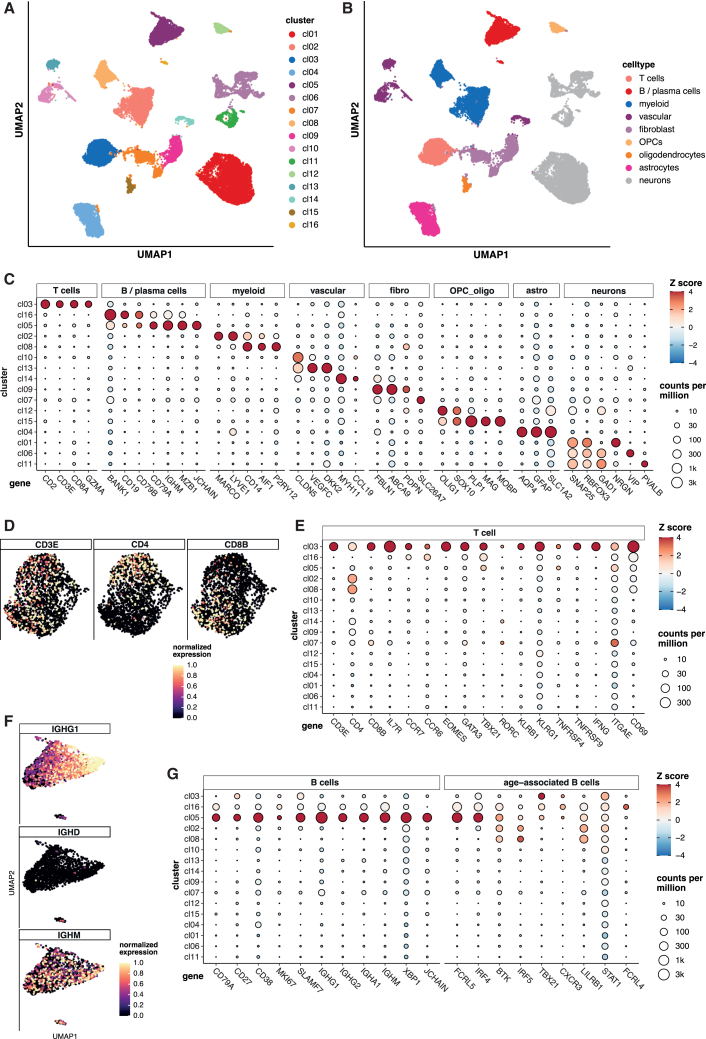
Single-nuclei RNA-seq reveals age-associated B cell phenotypes and IFN-γ^+^ T cells in inflamed meningeal MS tissue (A) UMAP of 24,939 single nuclei from highly inflamed meningeal and adjacent gray matter tissue sections (*n* = 3 MS, 2 biological replicate samples per donor and brain tissue block) and the 16 cell clusters identified by Harmony. Each dot corresponds to a single cell. (B) Annotation of the 16 clusters with respective labels for cell populations based on key marker genes. (C) Key phenotypic marker gene expression for all 16 clusters and their respective cell lineage annotations. The color code depicts the scaled mean log expression of the gene in each cluster. The mean expression of the respective gene in the cluster is denoted by the circle size. (D) UMAP of T cells with expression of *CD3E*, *CD4*, and *CD8*. Each dot corresponds to a single cell, colored by a gradient for expression level, which is scaled so that 0 equals zero expression and 1 equals 95^th^ percentile of log expression. (E) Relative and normalized expression levels of key characteristic T cell markers. (F) UMAP plot of B cells with expression of *IGHG1*, *IGHD*, and *IGHM*. (G) Relative and normalized expression levels of characteristic B cell, plasma cell, and age-associated B cell markers. See also Figure S1 and Tables S1 and S5.

We wondered whether the spontaneous and self-antigen-driven development of T-bet+CXCR3+ B cells and Th1 cells during AP may also be found in extrafollicular B-T cell structures in inflamed meninges of MS patients. We therefore performed single-nuclei RNA sequencing (snRNA-seq) on well-characterized brain tissue blocks with highly inflamed meningeal and adjacent gray matter tissue from three cases with secondary progressive MS (SPMS) to dissect cell phenotypes and gene signatures of CNS-infiltrating B and T cells. Biological replicates from the brain tissue of the same donors resulted in comparable sample quality and sufficient single-nuclei data (Figure S7). snRNA-seq analysis of the 24,939 isolated single nuclei revealed all relevant CNS-resident and immune cell populations, among them B and T cells, based on respective signature genes (Figures 7A, 7B, and S7 and Table S3). T cells were identified as cluster cl03, and two clusters, cl16 and cl05, were assigned to B cells. Cell numbers in cl16 were very low compared to cluster cl05, which expressed many signature genes for activated memory B cells and plasmablasts/cells (Figure 7C and Table S3). Markers of naive T and B cells were either underrepresented or not expressed, e.g., *IGHD* for B cells, indicating that snRNA-seq-derived data were not contaminated by blood-derived immune cells (Figures 7D–7G).

Among the T cells, we found both CD4^+^ and CD8^+^ T cells, and profiling of key genes showed expression of *TBX21* (T-bet) and *IFNG*, demonstrating that Th1/Tc1 phenotypes are prevalent among the meningeal T cell infiltrates. We were not able to detect high levels of chemokine receptors, which is attributable to the snRNA-seq technology that only captures nuclear RNA. However, we found various co-receptors such as *TNFRSF4* (OX-40) and *TNFRSF9* (4-1BB) that were also upregulated in AP+ T cells; markers such as *KLRG1*, *KLRB1*, and *EOMES*, characteristic of T cells with cytotoxic potential; and *ITGAE* (CD103) and *CD69* as marker genes of tissue-resident memory T cells (Figures 7D and 7E).

The infiltrating B cells showed strong expression of *IGHG1*, *IGHM*, *IGHA*, and *IGHG2* alongside *CD27* and *CD38*, which is consistent with antigen-experienced memory B cell/plasmablasts. Similar to AP, proliferating B cells were shown based on *MKI67* expression and genes usually upregulated in plasmablasts or activated B cells such as *SLAMF7*, *XBP1*, and *JCHAIN*. Further, meningeal B cells expressed several markers of ABCs like *TBX21*, low degree of *CXCR3* (technical limitation mentioned earlier), *FCRL5*, *FCRL4*, *LILRB1*, and IFN-γ driven genes (*IRF4*, *IRF5*, and *STAT1*) (Figures 7F and 7G). The aforementioned marker expression including the elevated *BTK* matches very well with the expression profile of AP+ B cells, suggesting that dominant B cell phenotypes in AP and meningeal inflammation share common features and that inflammation in the CNS compartment likely evolves from AP+ B and T cells in the peripheral immune system.

## Discussion

B-T cell interactions are fundamental for efficient immune protection and the GC response in SLOs. However, they may also result in B-T cell-rich ELS at chronically inflamed non-lymphoid tissue sites in autoimmune disorders such as MS.^41^ Interestingly, genetic risk loci associated with autoimmune diseases exhibit their strongest regulatory potential in cell populations involved in GC responses, underscoring the importance of dysregulated B-T cell responses in autoimmunity.^42^^,^^43^ ELS may range from simple lymphocyte aggregates to highly organized so-called tertiary lymphoid structures.^18^^,^^41^ ELS formation within meningeal cuffs adjacent to the brain and spinal cord gray matter have been linked with underlying cortical tissue pathology^5^^,^^19^ and also with chronic active lesions in the white matter in MS.^44^^,^^45^ Increasing evidence supports that ELS perpetuates chronic tissue inflammation and develops in response to disease-specific antigens. However, the mechanisms underlying the development of ectopic lymphoneogenesis are only partially understood.^41^ Difficulties in translating data from induced animal models and the limitations of analyzing frozen or fixed human brain tissue, which in MS usually only becomes available at the time of death, complicate the investigation of pathogenic B-T cell aggregates.

Using our human *in vitro* AP model, which is based on immunological synapse formation between pro-inflammatory B and T cells and involves self-peptide recognition through HLA-TCR contacts,^3^ we show here that the evolution of BTECs reflects many features of early-stage ELS formation during chronic tissue inflammation.^41^^,^^46^ We identified an activated AP+ B cell population as the main driver, which is expanded in MS and shows strong pro-inflammatory capacity, antigenic diversity through SHM, and maturation toward plasmablasts, which is unexpected due to the absence of exogenous stimuli. B cell activation and IFN-γ secretion also occur with anti-IgM stimulation in MS vs. HDs, however, without formation of BTECs. Activation of B cells differs between the two. AP depends on self-peptides presented by B cells and requires prolonged cell-cell interaction and T cell recognition, whereas anti-IgM directly stimulates B cells through the BCR. In MS, where B cells exhibit a more pro-inflammatory phenotype, anti-IgM reflects heightened B cell reactivity.

T-bet+CXCR3+ B cells are abundant in the AP+ compartment and also found in inflamed MS meninges. This concurs with data hinting at this subset as a major B cell phenotype in ELS in several autoimmune conditions including MS.^22^^,^^26^ CXCR3+ B cells accumulate in peripheral blood during early MS, during NAT treatment, and in CSF of MS patients.^23^^,^^24^^,^^47^^,^^48^^,^^49^ Most studies show that T-bet+ B cells are pro-inflammatory and contribute to the generation of pathogenic autoantibodies^26^^,^^50^ but also participate in humoral anti-viral responses by tightly interacting with Th1 cells within lymph nodes.^51^ Yet, knowledge about their role as antigen-presenting cells and their molecular and functional properties in MS has been limited. Stimulation through Toll-like receptor (TLR)7 or TLR9, IFN-γ, and interaction with T cells are key for inducing T-bet+ B cells.^52^^,^^53^ Here, we show the development of T-bet+ B and Th1 cells as a functional unit following AP, i.e., through the presentation of self-peptides and without TLR stimulus. Different from SLOs, where Tfh cells orchestrate B cell responses and maturation, AP involves classical and non-classical Th1 cells highly expressing PD-1 and ICOS, similar to pathogenic T cell phenotypes in rheumatoid arthritis.^54^

ELS formation has initially been reported to start with stromal cell and lymphoid tissue inducer cell interactions in the presence of lymphotoxin. Recent data suggest that immune cells such as B cells and other cytokines may substitute for these factors.^46^ Our data implicate a central role for IFN-γ in maintaining the inflammatory circuit and strong engagement within BTECs leading to the development of CXCR3+ B cells that further potentiate Th1 responses, a feedback loop that has been reported also in other autoimmune conditions.^55^ Our findings agree with data that spontaneously forming GCs during autoimmunity depend on IFN-γ and STAT1 signaling^32^ and that administration or expression of the Th1 cytokines tumor necrosis factor (TNF) and IFN-γ in the subarachnoid space of myelin oligodendrocyte glycoprotein (MOG)-immunized rats induces ELS formation in the meninges, subpial demyelination, and neuronal loss.^21^^,^^56^ JAK1/2 inhibition of AP by baricitinib further supports that IFN-γ is essential for BTECs and is currently investigated as treatment for rheumatoid arthritis.^57^ T-bet+ B and Th1 cells share several IFN-γ-induced genes, such as *TBX21*, *CXCR3*, *STAT1*, and *CXCL9*. Interestingly, CXCL9 has been proposed as a CSF biomarker in MS.^58^ It is upregulated in the meninges of postmortem brains with ELS formation^59^ hinting at B-T cell-related secretion in inflamed CNS tissue. Consistent with our findings, CXCL9 is expressed by CXCR3+ B cells and has been associated with a persistently active IFN-γ pathway and expansion of T-bet+ B cells.^60^ Another important aspect is the observation of T-bet+CXCR3+ B and T cells in meningeal tissue of patients, who show active inflammation and have advanced to the chronic stage of the disease (SPMS). This indicates that AP and B-T cell interactions not only are involved in the early relapsing-remitting disease but may also contribute to chronic inflammation in MS, which is thought to underlie progression independent of relapse activity.^61^

The hypomethylated sites for *STAT1*, *BTK*, *TBX21*, and *CXCR3* among others in AP+ B cells indicate a stable phenotype. It is well documented that B cells in MS display widespread hypomethylation.^62^^,^^63^ BTK may be overactive in T-bet+ B cells since BTK phosphorylation increases upon T-bet-inducing stimuli, whereas BTK inhibition reduces expression of T-bet.^33^ Consistent with this, we observed higher pBTK in AP+ B cells^3^ and prevention of BTECs in the presence of BTK inhibition. Whether BTK inhibition is as effective after BTECs formation remains to be addressed. We identified additional IFN-response genes that are upregulated in AP+ CXCR3+ B cells in MS, suggesting that IFN-γ is key in sensitizing B cells in MS. The resulting BTEC formation may then be sustained through additional interactions of co-receptor-ligand pairs, such as PD-1/PD-L1, 4-1BB/4-1BBL, and OX-40/OX-40L.

The development of AP+ CXCR3+ B cells into various functional memory phenotypes and plasmablasts may be driven through stem cell-like properties that have been reported for IgM+ T-bet+ B cells.^64^ Particularly the increased SHM in *IGHM* of AP+ B cells was unexpected since peripheral blood IgM+ B cells show usually low SHM. Interestingly, abundant SHM in *IGHM* was also observed in CSF-derived B cells of MS patients.^39^ Another parallel to CSF findings is the enrichment of *VH4*, in particular *VH4-34*+ BCRs in CSF,^38^^,^^39^^,^^40^ which we also found significantly increased in AP+ CXCR3+ B cells in MS. These data may be due to preferential recognition of specific antigens, including EBV or autoantigens like glial cell adhesion molecule (GlialCAM) and others.^38^ *VH4+* peripheral plasmablasts from MS patients preferentially recognized brain gray matter antigens,^65^ and a link between EBV and *VH4-34* has been hypothesized.^66^ In support, EBV reactivation is associated with the frequency of IgM+ T-bet+ memory B cells in MS patients.^67^ CXCR3+ memory B cells may be prone to develop into anti-EBNA1 IgG-secreting plasma cells,^68^ and anti-EBNA1 antibodies are associated with increased risk to develop MS, particularly in HLA-DR15+ patients.^8^ Anti-EBNA1 antibodies cross-recognize anoctamin 2 and GlialCAM, two recently identified MS target antigens.^38^^,^^69^ *De novo* EBV infection of B cells appears to lead to T-bet+ B cell development with upregulated pro-inflammatory signature genes and neuronal markers.^70^ The exacerbation of EAE upon infection with latent γHV68, a murine herpesvirus analog to EBV, also supports a role of T-bet+ B cells as drivers of neuroinflammation.^71^ These data raise the question whether EBV is involved in AP through the presence of EBV-transformed B cells and/or aberrant T cell reactivity to EBV.

Increased peripheral AP+ B cells are also associated with clinical findings including elevated CSF cell counts and higher numbers of spinal cord lesions. Both are associated with more active disease course, higher disability accrual after 10 years, higher risk of conversion from clinically isolated syndrome (CIS) to clinically definite MS, and an overall worse prognosis.^72^^,^^73^ Due to the short follow-up after sampling (4 years) and small sample size, it is not surprising that we did not find an association with clinical outcomes. The abundance of IgM in AP+ CXCR3+ B cells is interesting since lipid-reactive oligoclonal IgM antibodies have been linked with cognitive decline^74^ and increased brain atrophy^75^ and to indicate a worse disease evolution in MS,^76^ and AP+ CXCR3+ B cells may be involved in this process.

Our data provide an in-depth characterization of the interactions between two key cell types with highly complementary phenotypes, T-bet+CXCR3+ B cells and T-bet+ CXCR3+ IFN-γ-secreting Th1 cells. BTECs start their interactions, and B cells are probably most important for initiating, amplifying, and sustaining the inflammatory response, the antigen presentation, and attraction to the CNS compartment, while T cell-derived IFN-γ enhances HLA-class II expression, immune synapse formation, AP, and the recognition of MS autoantigens, which may all contribute to inflammation in the brain. The Th1-derived IFN-γ pathway appears to be at the core of MS pathogenesis. Administration of IFN-γ in a clinical trial worsened MS.^77^ IFN-γ has been shown to lead to synapse loss^78^ and to cause hypomyelination, reactive gliosis,^79^ and upregulation of major histocompatibility complex class II on several brain cell types including neurons and oligodendrocytes,^80^^,^^81^ which renders the latter targets for CD4^+^ T cells. Hence, the IFN-γ-mediated effects extend far beyond the interactions of B and T cells and also shape the local inflammation in the brain, likely during both early and late stages of the disease. We anticipate that our *in vitro* findings about BTEC formation, expansion of T-bet+CXCR3+ B cells, hyperreactive B and T cell AP,^3^ the involved genetic and environmental risk factors,^4^ and how these translate into MS pathomechanisms not only may guide further studies of the genetic and environmental contributions but will also be instrumental for testing existing and developing new treatments for MS.

### Limitations of the study

Apart from meningeal tissue analyses, our study is limited to an *in vitro*-based disease model. Yet it allowed to assess the functional relationship and phenotypic evolution of pro-inflammatory B-T cell interactions in MS patients. The link to clinical observations is based on associations and should be expanded by longer follow-up, larger group sizes, and paired PBMC and CSF. The number of therapy-naive PMS blood samples was limited rendering meaningful statistics challenging. Furthermore, no early-stage brain tissue could be examined, which will be important in the future to assess if AP and pro-inflammatory B-T cell interactions start in the CNS compartment already early.

## Resource availability

### Lead contact

Further information and requests for resources and reagents should be directed to the lead contact, Prof. Dr. Roland Martin (roland.martin@uzh.ch).

### Materials availability

This study did not generate new unique reagents.

### Data and code availability

• The published article includes all data that had been generated or analyzed during this study and summarized in the accompanying tables, figures, and supplemental information. RNA sequencing data of AP+ B cells and CD4^+^ T cells have been deposited in the European Nucleotide Archive (ENA). Transcriptome and methylome, scRNA-seq, and snRNA-seq raw data reported in this paper have been deposited in the European Genome-Phenome Archive (EGA). All accession numbers are listed in the key resources table.
• This paper does not report original code.
• Any additional information required to reanalyze the data reported in this paper is available from the lead contact upon request.

## Acknowledgments

This study was supported by 10.13039/501100000781European Research Council grant ERC-2013-ADG 340733 to R.M. and ERC-CoG 818170 to M.J., the Clinical Research Priority Program MS (CRPP^MS^) of the University of Zurich (UZH), 10.13039/501100001711Swiss National Science Foundation (SNF) Sinergia grant UnmetMS, the 10.13039/501100004063Knut and Alice Wallenberg Foundation, the Swedish MS Research Foundation, and the Swedish Brain Foundation. Single-cell and single-nuclei RNA-seq were funded by F. Hoffmann-La Roche. We thank J. Duarte, N. Vilarrasa, D. Istanbullu, P. Tomas, J. Rex (NIMS, University Hospital Zurich, USZ), L. Fuentes Font (NIMS, USZ, and Imperial College), R. Schlapbach, A. Bratus, and L. Opitz (Functional Genomics Center Zurich) for expert and/or technical support; M. Manz and A. Müller (USZ) for leukaphereses; M. Foege for regulatory and K. Léger for science administration support; and T. Eng and the staff of the MS Outpatient Clinic and Day Hospital (Neurology Clinic, USZ) for patient care-related aspects. We acknowledge the National Genomics Infrastructure (NGI) in Stockholm for assistance with Illumina sequencing. We thank J. Trück (UZH) and C. Münz (UZH) for their helpful comments.

## Author contributions

Ivan Jelcic and R.M. designed the overall study and experiments, interpreted the data, and wrote the manuscript. Ivan Jelcic, R.N., Z.M., and F.A.N. performed and analyzed AP experiments and interpreted the data. D.C. processed samples for scRNA-seq and snRNA-seq. W.M. analyzed snRNA-seq data. Ivan Jelcic, W.M., C.R., and R.R. interpreted the data. I.F., D.M., and M.D.R. analyzed the scRNA-seq data, performed statistical analyses, interpreted the data, and contributed to manuscript writing. C.E., J.S.d.C., and L.P. used 4i technology and interpreted the data. Y.H., G.Z., and M.J. processed, analyzed, and interpreted transcriptome and methylome data and contributed to manuscript writing. R.R. characterized and selected inflamed brain/meningeal tissue. D.E. and V.K. processed meningeal tissue. M.K. and F.P. provided rituximab-treated MS patient samples. Ilijas Jelcic, I.R., and R.M. organized sample collection and provided clinical data characterization. All authors revised the manuscript.

## Declaration of interests

R.M. has received unrestricted grants from Biogen, Novartis, Roche, and Third Rock and honorary for advisory roles and lectures for Roche, Novartis, Biogen, Genzyme, Neuway, CellProtect, Third Rock, and Teva. He is a patent holder and co-holder on patents of daclizumab in MS (held by the NIH), JCV VP1 for vaccination against PML, JCV-specific neutralizing antibodies to treat PML, antigen-specific tolerization with peptide-coupled cells, novel autoantigens in MS, and designer neoantigens for tumor vaccination (all held by the University of Zurich). He is a co-founder of Abata Therapeutics, Watertown, MA, USA, and co-founder and employee of Cellerys AG, Schlieren, Switzerland. Ilijas Jelcic has received speaker honoraria or unrestricted grants from Biogen Idec and Novartis and received compensation for advice or lecturing for Alexion, Biogen, Bristol Myers Squibb, Celgene, Janssen-Cilag, Neuway, Merck, Novartis, Roche, and Sanofi Genzyme; none of these are related to this study. Ivan Jelcic, W.M., D.C., C.R., B.T., and D.M. are or have been employees of F. Hoffmann-La Roche. V.K. received travel and/or speaker honoraria from and/or served on advisory boards for Biogen, Merck, Novartis, and Roche; none of these are related to this study. Z.M., P.O., M.S., and R.M. are employees of Cellerys AG, Switzerland. I.F. is the founder of YugaCell.

## STAR★Methods

### Key resources table

**Table:** 

REAGENT or RESOURCE	SOURCE	IDENTIFIER
AIM-V medium	Thermo Fisher Scientific	Cat# 12055-091
Fetal calf serum (FCS)	Eurobio	Cat# CVFSVF0001
Goat anti-IgM antibody	Jackson ImmunoResearch	Cat# 109-006-129 RRID:AB_2337553
Human serum (from 10 donors, AB male, pooled and heat inactivated)	Basel blood donation center	NA, order specific
IgG from human serum	Sigma-Aldrich	Cat# I2511; RRID:AB_1163604
IMDM medium	Cytiva	Cat# SH30259.01
Rat anti-human AID (clone EK2-5G9), (FACS, *in vitro)*	BD Biosciences	Cat# 565785; RRID:AB_2739355
Rabbit anti-CD4 (clone EPR6855), (4i, *in vitro*)	Abcam	Cat#ab133616; RRID:AB_2750883
Mouse anti-CD20 (clone L26), (4i, *in vitro*)	Dako	Cat# M0755; RRID:AB_2282030
Rabbit anti-phospho-p44/42 MAPK (ERK 1/2) pT202/pY204; (4i, *in vitro*)	Cell Signaling Technology	Cat# 9101; RRID:AB_331646
Rabbit anti-PCNA, (4i, *in vitro*)	Abcam	Cat# ab139696; RRID:AB_2894712
Rabbit anti-phospho-Akt (clone D9E); (4i, *in vitro*)	Cell Signaling Technology	Cat#4060; RRID; AB_2315049
Mouse anti-HLA-DP/DQ/DR (clone CR3/43); (4i, *in vitro*)	Dako	Cat# M0775; RRID:AB_2313661
Rabbit anti-Zap-70 (pY319)/Syk (pY352); (4i, *in vitro*)	Cell Signaling Technology	Cat# 2701; RRID:AB_331600
Mouse anti-NFkappaB p65 (clone F-6); (4i, *in vitro*)	Santa Cruz Biotechnolpgy	Cat# sc-8008; RRID:AB_628017
Mouse anti-CD3 (clone HIT3a); (FACS, *in vitro*)	Biolegend	Cat# 300328; RRID:AB_1575008
Mouse anti-CD3 (clone OKT3); (FACS, *in vitro*)	Biolegend	Cat# 317328; RRID:AB_2562907
Mouse anti-CD3 (clone UCHT1); (FACS, *in vitro*)	Biolegend	Cat# 300423; RRID:AB_493740
Mouse anti-CD4 (clone OKT4); (FACS, *in vitro*)	Biolegend	Cat# 317438; RRID:AB_11218995
Mouse anti-CD4 (clone OKT4); (FACS, *in vitro*)	Biolegend	Cat# 317418; RRID:AB_571947
Mouse anti-CD8 (clone SK1); (FACS, *in vitro*)	Biolegend	Cat# 344718; RRID:AB_10551438
Mouse anti-CD8 (clone DK25); (FACS, *in vitro*)	Dako	Cat# PB98401; RRID:AB_579530
Mouse anti-CD19 (clone HIB19); (FACS, *in vitro*)	Biolegend	Cat# 302226; RRID:AB_493751
Mouse anti-CD19 (clone HIB19); (FACS, *in vitro*)	Biolegend	Cat# 302212; RRID:AB_314242
Mouse anti-CD19 (clone SJ25C1); (FACS, *in vitro*)	Biolegend	Cat# 363006; RRID:AB_2564128
Mouse anti-CD20 (clone 2H7); (FACS, *in vitro*)	Biolegend	Cat# 302306; RRID:AB_314254
Mouse anti-CD27 (clone M-T271); (FACS, *in vitro*)	Biolegend	Cat# 356406; RRID:AB_2561825
Mouse anti-CD27 (clone M-T271); (FACS, Legendscreen and *in vitro*)	Biolegend	Cat# 356412; RRID:AB_2562258
Mouse anti-CD28 (clone CD28.2); (FACS, *in vitro*)	Biolegend	Cat# 302908; RRID:AB_314310
Mouse anti-CD38 (clone HIT2); (FACS, *in vitro*)	Biolegend	Cat# 303515; RRID:AB_1279235
Mouse anti-CD45 (clone HI-30) (FACS, Legendscreen)	Biolegend	Cat# 304012; RRID:AB_314400
Mouse anti-CD45 (clone HI-30) (FACS, Legendscreen)	Biolegend	Cat# 304014; RRID:AB_314402
Mouse anti-CD45 (clone HI-30) (FACS, Legendscreen)	Biolegend	Cat# 304028; RRID:AB_893338
Mouse anti-CD45RA (clone HI100); (FACS, *in vitro*)	Biolegend	Cat# 304112; RRID:AB_314416
Mouse anti-CD56 (NCAM) (clone 5.1H11); (FACS, *in vitro*)	Biolegend	Cat# 362544; RRID:AB_2565922
Mouse anti-CD134 (OX40) (clone Ber-ACT35); (FACS, *in vitro*)	Biolegend	Cat# 350007; RRID:AB_10722613
Mouse anti-CD137 (4-1BB) (clone 4B4-1); (FACS, *in vitro*)	Biolegend	Cat# 309804; RRID:AB_314783
Mouse anti-CD137 (4-1BB) (clone 4B4-1); (FACS, *in vitro*)	Biolegend	Cat# 309809; RRID:AB_830671
Mouse anti-CD138 (clone MI15); (FACS, *in vitro*)	Biolegend	Cat# 356509; RRID:AB_2561897
Mouse anti-CD154 (CD40L) (clone 24–31); (FACS, *in vitro*)	Biolegend	Cat# 310809; RRID:AB_314832
Mouse anti-CD183 (CXCR3) (clone G025H7); (FACS, *in vitro*)	Biolegend	Cat# 353706; RRID:AB_10962912
Mouse anti-CD183 (CXCR3) (clone G025H7); (FACS, Legendscreen)	Biolegend	Cat# 353716; RRID:AB_2561448
Mouse anti-CD185 (CXCR5) (clone J252D4); (FACS, *in vitro*)	Biolegend	Cat# 356935; RRID: AB_2629527
Mouse anti-CD196 (CCR6) (clone G034E3); (FACS, *in vitro*)	Biolegend	Cat# 353416; RRID:AB_10915987
Mouse anti-CD197 (CCR7) (clone G043H7); (FACS, *in vitro*)	Biolegend	Cat# 353226; RRID:AB_11126145
Mouse anti-CD278 (ICOS) (clone ISA-3); (FACS, *in vitro*)	Thermo Fisher Scientific	Cat# 17-9948-41; RRID:AB_10597585
Mouse anti-CD279 (PD-1) (clone NAT105); (FACS, *in vitro*)	Biolegend	Cat# 367425; RRID:AB_2721544
Mouse anti-CXCL9 (clone J1015E10); (FACS, *in vitro*)	Biolegend	Cat# 357905; RRID:AB_2566033
Mouse anti-HLA-DR (clone L243); (FACS, *in vitro*)	Biolegend	Cat# 307606; RRID:AB_314684
Mouse anti-HLA-DR (clone L243); (FACS, *in vitro*)	Biolegend	Cat# 307616; RRID:AB_493588
Mouse anti-IgD (clone IA6-2); (FACS, *in vitro*)	Biolegend	Cat# 348222; RRID:AB_2561595
Mouse anti-IgM (clone G20-127); (FACS, *in vitro*)	BD Biosciences	Cat# 562618; RRID:AB_2737681
Mouse anti-IgG (clone G18-145); (FACS, *in vitro*)	BD Biosciences	Cat# 561297; RRID:AB_10611877
Mouse anti-STAT1 (pY701) (clone 4a); (FACS, *in vitro*)	BD Biosciences	Cat# 612564; RRID:AB_399855
Mouse anti-STAT4 (pY693) (clone 38/p-Stat4); (FACS, *in vitro*)	BD Biosciences	Cat# 558137; RRID:AB_397052
Mouse anti-STAT5 (pY694) (clone 47/Stat5); (FACS, *in vitro*)	BD Biosciences	Cat# 560117; RRID:AB_1645546
Mouse anti-T-bet (clone 4B10); (FACS, *in vitro*)	Biolegend	Cat# 644813; RRID:AB_10896913
Mouse isotype control (clone MOPC-21; IgG1, κ); (FACS, *in vitro*)	Biolegend	Cat# 400123
Mouse isotype control (clone MOPC-173; IgG2a, κ); (FACS, *in vitro*)	Biolegend	Cat# 400223
Mouse isotype control (clone MPC-11; IgG2b, κ); (FACS, *in vitro*)	Biolegend	Cat# 400323
Rat isotype control (clone LOU; IgG2b, κ); (FACS, *in vitro*)	BD Biosciences	Cat# 557691
RPMI-1640 medium	Sigma-Aldrich	Cat# R0883
X-Vivo medium	Lonza	Cat# BE04-418F

**Biological samples**		

Cerebrospinal fluid (CSF)	*This paper*	*Neuroimmunology and MS Research, Neurology Clinic, University Hospital, Zurich, Switzerland*
Peripheral blood	*This paper*	*Neuroimmunology and MS Research, Neurology Clinic, University Hospital, Zurich, Switzerland*
Peripheral blood	*This paper*	*Neuroimmunology and Clinical MS Research, University Medical Center Eppendorf, Hamburg, Germany*
Peripheral blood	*This paper*	*Department of Neurology, Karolinska University Hospital, Stockholm, Sweden*

**Chemicals, peptides, and recombinant proteins**		

N-acetyl-L-cysteine	Sigma-Aldrich	Cat# A9165
Bovine serum albumin (BSA)	Roth	Cat# 3854.3
Baricitinib (INCB028050)	Selleckchem	Cat# S2851
N-acetyl-L-cysteine	Sigma-Aldrich	Cat# A9165
Carboxyfluorescein diacetate N-succinimidyl ester (CFSE)	Sigma-Aldrich	Cat# 21888
DAPI (4′,6-Diamidino-2-Phenylindole, Dihydrochloride)	Thermo Fisher Scientific	Cat# D1306; RRID:AB_2629482
Dimethyl sulfoxide (DMSO)	Applichem	Cat# A3672
DNAse I, recombinant	Roche	Cat# 04536282001
Exonuclease I	New England Biolabs	Cat# M0293S
Ficoll	Eurobio	Cat# GAUFIC0065
G418 sulfate (geneticin)	Thermo Fisher Scientific	Cat# 10131035
Gentamicin	Sigma-Aldrich	Cat# G1397
HEPES	Gibco	Cat# 15630-080
Ibrutinib (PCI-32765; BTK inhibitor)	Selleckchem	Cat# S2680
Klenow exo	New England Biolabs	Cat# M0212L
L-glutamine	Thermo Fisher Scientific	Cat# 25030-081
LIVE/DEAD™ Fixable Aqua Dead Cell Stain Kit	Thermo Fisher Scientific	Cat# L34957
LIVE/DEAD™ Fixable Yellow Dead Cell Stain Kit	Thermo Fisher Scientific	Cat# L34959
LIVE/DEAD™ Fixable Near-IR Dead Cell Stain Kit	Thermo Fisher Scientific	Cat# L10119
LIVE/DEAD™ Fixable Violet Dead Cell Stain Kit	Thermo Fisher Scientific	Cat# L34955
Maleimide	Sigma-Aldrich	Cat# 129585
Methanol	Sigma Aldrich	Cat# 322415
Paraformaldehyde, EM Grade, Purified (Polyoxymethylene)	Electron Microscopy Sciences	Cat# 15710
Penicillin/Streptomycin	Corning	Cat# 30-002-Cl
Phytohemagglutinin-L (PHA-L)	Sigma-Aldrich	Cat# L2769
Proteinase K, recombinant	Roche	Cat# 03115879001
QIAzol lysis reagent	Qiagen	Cat# 79306
Saponin	Applichem	Cat# A4518
Succinimidyl Ester, Alexa Fluor™ 647 NHS Ester	Thermo Fisher Scientific	Cat# A20006
Tris (2-Carboxyethyl) phosphine Hydrochloride	Ubiquitin-Proteasome Biotechnologies	Cat# P1020-25
Triton X-100	Sigma-Aldrich	Cat# T8787

**Critical commercial assays**		

CD4 T cell Isolation Kit, human (untouched)	Miltenyi Biotec	Cat# 130-096-533
CD19 MicroBeads, human	Miltenyi Biotec	Cat# 130-050-301
CD20 MicroBeads, human	Miltenyi Biotec	Cat# 130-091-104
Cytofix/Cytoperm	BD Biosciences	Cat# 554714
CXCL9 DuoSet ELISA	R&D Systems	Cat# DY392-05
Foxp3/Transcription Factor Staining Buffer Set	Thermo Fisher Scientific	Cat# 00-5523-00
GolgiStop protein transport inhibitor	BD Biosciences	Cat# 554724
LEGENDplex™ Th Cytokine Panel	Biolegend	Cat# 740001
LEGENDScreen™ Human PE Kit	Biolegend	Cat# 700007
PicoPure RNA Isolation Kit	Thermo Fisher Scientific	Cat# KIT0204
SMARTer stranded total RNA-seq- pico input mammalian kit	Clontech	Cat# 635007
TruSeq SR Cluster Kit v4-cBot-HS	Illumina	Cat# GD-401-4001
Chromium Next GEM Single Cell 5′ Library & Gel Bead Kit v1.1	10x Genomics	Cat# PN-1000167
Chromium Single Cell 5′ Library Construction Kit	10x Genomics	Cat # PN-1000020
Chromium Single Cell V(D)J Enrichment Kit Human B cells	10x Genomics	Cat # PN1000016
Chromium Next GEMChip G Single Cell Kit	10x Genomics	Cat # PN-1000120
Chromium i7 Multiplex Kit	10x Genomics	Cat #PN-120262
NovaSeq 6000 S2 Reagent Kit v1.5 (300 cycles)	Illumina	Cat # 20028314
dsDNA Quantitation, high sensitivity	Thermo Fisher Scientific	Cat #Q32854
High Sensitivity D1000 ScreenTape	Agilent Technologies	Cat # 5067-5584
High sensitivity D1000 Reagents	Agilent Technologies	Cat # 5067-5585
Imprint DNA Modification Kit	Sigma	Cat# MOD50
Agencourt Ampure XP beads	Beckman Coulter	Cat# A63880
M-280 Streptavidin Dynabeads	Invitrogen	Cat# 11205D
KAPA HiFi HotStart DNA polymerase	Roche	Cat# KK2501
KAPA Library Quantification Kit	Roche	Cat# KK4824
High Sensitivity DNA Analysis Kit	Agilent	Cat# 5067-4626
Direct-zol™RNA MicroPrepKit	Zymo	Cat# R2070T

**Deposited data**		

RNA sequencing data of non-proliferating and autoproliferating B cells	Jelcic et al.*,**^3^* 2018 *and this paper*	ENA: PRJEB23143
RNA sequencing data of non-proliferating and autoproliferating CD4^+^ T cells	Jelcic et al.*,**^3^* 2018 *and this paper*	ENA: PRJEB19652
Single cell sequencing (RNA + VDJ) of non-proliferating and autoproliferating B cells	*this paper, reposited in EGA*	EGA dataset: EGAD50000001237; EGA study: EGAS50000000845
Single nuclei RNA sequencing of MS meningeal tissue	*this paper,* *reposited in EGA*	EGA dataset: EGAD50000001235; EGA study: EGAS50000000845
Transcriptome of non-proliferating and autoproliferating memory B cells	*this paper,* *reposited in EGA*	EGA dataset: EGAD50000001270; EGA study: EGAS50000000872
Methylome of non-proliferating and autoproliferating memory B cells	*this paper,* *reposited in EGA*	EGA dataset: EGAD50000001269; EGA study: EGAS50000000872

**Oligonucleotides**		

Oligo 1 (CTACACGACGCTCTTCCGATCTNNNNNNNN-N)	Biotin	

**Recombinant DNA**		

*not in this study*		

**Software and algorithms**		

EAGLE2	Loh et al., 2016 ^82^	https://data.broadinstitute.org/alkesgroup/Eagle/
FlowJo	Tree Star	https://www.flowjo.com/; RRID:SCR_008520
GraphPad Prism	Graphpad	http://www.graphpad.com/; RRID:SCR_002798
Ingenuity Pathway Analysis	Qiagen	http://www.ingenuity.com/; RRID:SCR_008653
PBWT	Durbin et al., 2014 ^83^	https://github.com/richarddurbin/pbwt
PRSice v1.25	Euesden et al., 2015 ^84^	http://prsice.info/
R/bioconductor package edgeR	Robinson et al., 2009^85^	http://bioconductor.org/packages/release/bioc/html/edgeR.html
RSEM algorithm (version 1.2.22)	Li and Dewey, 2011 ^86^	http://omictools.com/; RRID:SCR_002250
Trimmomatic	Bolger et al., 2014 ^87^	http://www.usadellab.org/cms/index.php?page=trimmomatic; RRID:SCR_011848
TissueMAPs	Pelkmans lab	
Cellpose	Stringer et al.,^88^ 2021	
Spatstat	Baddeley and Turner,^89^ 2005	www.jstatsoft.org

***Paired methylome and transcriptome***		

Trim Galore (0.6.10)	The Babraham Institute	https://github.com/FelixKrueger/TrimGalore
Bismark (0.24.0)	Krueger et al., 2011 ^90^	https://www.bioinformatics.babraham.ac.uk/projects/bismark/
Limma (v3.48.3)	Ritchie et al.,^91^ 2015	https://bioconductor.org/packages/release/bioc/html/limma.html
Nfcore/rnaseq (v1.0)	Ewels et al., 2020 ^92^	https://nf-co.re/rnaseq/1.0
Deseq2 (v1.32.0)	Love et al., 2014 ^93^	https://bioconductor.org/packages/release/bioc/html/DESeq2.html
GSEA (v4.1.0)	Subramanian et al., 2005 ^94^	https://www.gsea-msigdb.org/gsea/index.jsp

***scRNAseq***		

Alevin/Salmon	Srivastava et al., 2019 ^95^	https://salmon.readthedocs.io/en/latest/alevin.html
AnnotationDbi	Pagès et al., 2024 ^96^	https://bioconductor.org/packages/AnnotationDbi
CellID	Cortal et al., 2019 ^97^	https://github.com/cbl-imagine/CellID
Conos	Barkas et al., 2019 ^98^	https://github.com/kharchenkolab/conos
data.table		https://CRAN.R-project.org/package=data.table
destiny	Angerer et al., 2015 ^99^	http://bioinformatics.oxfordjournals.org/content/32/8/1241
e1071		https://CRAN.R-project.org/package=e1071
ggplot2		https://ggplot2.tidyverse.org
ggrepel		https://CRAN.R-project.org/package=ggrepel
ica		https://CRAN.R-project.org/package=ica
leiden	Traag et al., 2019 ^100^	https://github.com/TomKellyGenetics/leiden
Magrittr		https://CRAN.R-project.org/package=magrittr
Muscat	Crowell et al., 2020 ^101^	https://github.com/HelenaLC/muscat
nlme		https://CRAN.R-project.org/package=nlme
pheatmap		https://CRAN.R-project.org/package=pheatmap
ROCR	Sing et al., 2005 ^102^	http://rocr.bioinf.mpi-sb.mpg.de
Rsamtools	Morgan et al., 2020 ^103^	https://bioconductor.org/packages/Rsamtools
scDblFinder	Germain et al., 2021 ^104^	https://github.com/plger/scDblFinder
sctransform	Hafemeister et al.,^105^ 2019	https://doi.org/10.1186/s13059-019-1874-1
SingleCellExperiment	Amezquita et al., 2020 ^106^	https://www.nature.com/articles/s41592-019-0654-x
umap		https://CRAN.R-project.org/package=umap

**Other**		

Cell culture microplate 384 well, F-bottom, Microclear, black, TC, sterile	Greiner	Cat# 781902
Lid, PS, Low profile (6 mm), clear, sterile	Greiner	Cat# 656191

### Experimental model and study participant details

#### Human subjects

Peripheral blood or leukapheresis was collected in a first cohort from 32 healthy donors (HD; age range 25–49, F:M ratio: 1.7) and 53 untreated (nihil) patients with relapsing-remitting MS (RRMS), of which 18 patients where in relapse (REL; age range 20–45, F:M ratio: 2.0) and 35 patients were in remission (REM; age range 22–54, F:M ratio: 1.1) (see also Jelcic et al., 2018^3^), as well as 13 untreated patients with progressive MS (PMS; age range 26–65, F:M ratio: 0.9). Patients did not receive glucocorticoids for at least 4 weeks and no other immunomodulatory treatments for at least 12 weeks before blood sampling. Furthermore, peripheral blood was collected from 15 patients with RRMS under natalizumab treatment (age range 23–50, F:M ratio: 4.0) and from 14 patients with RRMS under rituximab treatment (age range 30–59, F:M ratio: 1.3) (see also Jelcic et al., 2018^3^). In an additional cohort, we used peripheral blood from 13 HD (age range 25–41, F:M ratio: 0.9), 16 untreated RRMS patients (REM; age range 22–54, F:M ratio: 1.3) and 5 untreated PMS patients (age range 47–65, F:M ratio: 1.5) to analyze CXCR3+ B cells in AP. CSF samples for some of the above MS patients have been taken for routine diagnostic purposes. Peripheral blood was collected from 6 patients with RRMS under natalizumab treatment (age range 23–34, only F) for parallel downstream transcriptome and methylome profiling of pre-sorted AP B cells. Peripheral blood or leukapheresis from 5 HD (age range 20–49, F:M ratio: 1.5), 6 untreated RRMS patients (REM; age range 22–48, F:M ratio: 2.0) and 5 RRMS patients under natalizumab treatment (age range 23–42, F:M ratio: 1.5) was collected for downstream application of scRNAseq from pre-sorted AP+ B cells. MS had clinically definite MS by clinical and/or McDonald criteria^107^, and relapse was defined as clinical worsening of at least 24 h duration and/or contrast-enhancing lesion/s on MRI and within no more than one month of peripheral blood collection. Remission was defined as absence of contrast-enhancing lesion/s on MRI and stable clinical disease course compared to last visit as well as being at least one month before and/or after relapse. The majority of samples were collected at the Neuroimmunology and MS Research Section, Neurology Clinic, University Hospital Zurich, Zurich (Switzerland), and some samples from the first cohort at the Institute for Neuroimmunology and Clinical Multiple Sclerosis Research, Center for Molecular Neurobiology, University Medical Center Eppendorf, Hamburg (Germany). The demographics of all donors included in the study are shown in detail in Table S1. Please note that in our previous study,^3^ the same patient samples were used primarily for analysis of AP+ T cells and only partially also for AP+ B cells as shown in Figure 3 of Jelcic et al., 2018.^3^ Yet, the B cell data that were also acquired with the same patient sample were not analyzed to the extent (e.g., CXCR3, patient groups, molecular profiling) as provided in this present manuscript, while we also added additional patient samples. No detailed exploration of the dataset with regard to AP+ B cells was performed in the previous study^3^, and all analyses and conclusions of the present study are completely independent.

The samples used for the research activities were taken from various research projects. These have been previously reviewed and approved by the corresponding Ethics Committees. This includes: **a**) research projects with EC-No. 2758 approved on 18^th^ October 2007 by the Ethics Committee of the Hamburg Board of Physicians, Germany; **b**) research projects with EC-No. 2013-0001 approved on 05^th^ June 2013 and EC-No. 2014-0699 approved on 27^th^ February 2015 by the Cantonal Ethics Committee of Zurich, Switzerland; **c**) research project with EC-No. 2015/1280-32 approved on 21st July 2015 by Ethical Vetting Board of Stockholm, Sweden; **d**) brain tissue samples were provided for snRNAseq by the UK Multiple Sclerosis Society Tissue Bank. The use of these samples for research projects was approved respectively by the Regional Ethics Committee for Wales, UK, on 18^th^ June 2013 (EC-No. 08/MRE09/31 + 5) and by the Cantonal Ethics Committee of Zurich, Switzerland, on 4^th^ September 2014 (EC-No. 2014-0243). All patients consented for the sampling in the framework of the above-mentioned projects. In addition, all of these patients have consented for the further use of the samples in research (General Consent).

### Method details

#### Peripheral blood mononuclear cells

Peripheral blood mononuclear cells (PBMCs) from HD and patients were freshly isolated from EDTA-containing blood tubes or from leukaphereses using Ficoll (Eurobio) density gradient centrifugation. All isolated PBMCs were cryopreserved in freezing media containing 10% dimethyl sulfoxide (DMSO; Applichem) and 90% fetal calf serum (FCS; Eurobio) and stored at −180°C. PBMCs were obtained under ethical approvals and with informed consent as described above. The demographics incl. age and gender of these donors in the respective cohorts are listed in Table S1.

#### Serum and cerebrospinal fluid

Paired serum and CSF samples were collected from 24 RRMS patients during routine diagnostic work-up. Peripheral blood for testing AP was collected within 2 years after lumbar puncture. All patients were therapy-naïve both at peripheral blood collection and at lumbar puncture. Routine CSF parameters included: CSF cell count, total protein, CSF/serum albumin ratio (Q_alb_), intrathecal synthesis of IgA, IgM, and IgG including oligoclonal IgG bands (OCB). A CSF cell count >5/μL was classified as ‘increased’. Protein, albumin and total IgG, -IgM, and -IgA in serum and CSF were quantified by immunonephelometry (Siemens Healthineers, Zürich, Switzerland). IgG index was calculated as IgG index = Q_IgG/_Q_alb_ (with Q_IgG_ = IgG_CSF_/IgG_serum_ and Qalb = alb_CSF_/alb_serum_) and IgM index and IgA index each accordingly. IgG index ≥0.7 indicated intrathecal synthesis of IgG. The relative intrathecal fraction of IgG, IgM and IgA (IgG_IF_, IgM_IF_ and IgA_IF_), were calculated according to Reiber.^108^ IgG_IF_ ≥ 10%, IgM_IF_ ≥ 10% and IgA_IF_ ≥ 10% indicated significant intrathecal synthesis of IgG, IgM and IgA, respectively. OCBs were detected by isoelectric focusing on agarose gels and immunoblotting using IgG-specific antibodies (SEBIA Swiss GmbH, Wollerau, Switzerland) in a semi-automated analyzer (EasyFix Interlab G26, Apteq AG, Cham, Switzerland), and CSF-specific OCBs were considered positive in case of ≥2 additional bands.

#### Clinical parameters

We assessed female/male ratio, age at disease onset, age at sampling of peripheral blood for testing AP, age at last follow-up, disease duration and other clinical parameters including expanded disability status scale (EDSS), multiple sclerosis severity score (MSSS) according to Roxburgh et al.^109^ and age-related MS severity scale (ARMSS) according to Manouchehrinia et al.,^110^ as well as the history of optic neuritis, spinal syndrome or brain stem syndrome. We also assessed the prevalence and number of spinal cord lesions and brain stem lesions on MRIs at sampling and at follow-up.

#### Autoproliferation and control stimulations

Autoproliferation (AP) assay and IgM stimulation were performed as previously described.^3^ Cryopreserved PBMCs were thawed with complete IMDM medium and afterward washed once with serum-free AIM-V medium (Thermo Fisher Scientific). For assessment of AP in flow cytometry, cells were labeled with carboxyfluorescein diacetate N-succinimidyl ester (CFSE) whereas for the 4i procedure cells were kept unstained. For CFSE staining cells were washed twice with PBS containing 0.1% human serum (HS; blood bank Basel), resuspended at a concentration of 10 × 10^6^ cells/ml in PBS/0.1% HS and were then labeled at a final concentration of 0.5 μM CFSE (Sigma-Aldrich) for 3 min at room temperature. The labeling was stopped by quenching with 5x excess volume of cold complete RPMI medium containing 10% HS. After one further wash step with AIM-V, CFSE-labeled cells were seeded at 2 × 10^5^ PBMCs/200 μL per well in AIM-V (10–12 replicate wells per donor and condition) in 96-well U-bottom microtiter plates (Greiner Bio-One) at 37°C, 5% CO_2_, in the absence of exogenous stimuli for 7 days (AP).

For conventional T cell reactions, we used for the same donors as control PHA (0.5 μg/mL) As conventional B cell reaction, we stimulated PBMCs by cross-linking with an anti-human IgM antibody (10 μg/mL, Jackson ImmunoResearch). We also analyzed AP in the presence of BTK inhibitor (ibrutinib) and JAK-STAT inhibitors (tofacitinib and baricitinib) (key resources table).

#### B-T cell co-cultures

Naive and CXCR3-or CXCR3+ memory B cells were sorted through the Sony SH800 cell sorter upon labeling PBMCs from MS patients with antibodies against CD19, CD27 and CXCR3. Sorted cells were subsequently put in AIM-V medium and adjusted to a concentration of 5 × 10^5^cells per mL before co-culturing with autologous T cells. In parallel, PBMCs from the same donors were labeled with CFSE as described above and used to magnetically isolate untouched autologous CD4^+^ T cells according to the manufacturer's instructions (Miltenyi Biotec). All cell populations were checked for at least 90% purity in flow cytometry. Isolated CFSE-labeled CD4^+^ T cells (5 × 10^4^ cells) were cultured either alone, with sorted autologous naive, CXCR3- or CXCR3+ memory B cells (5 × 10^4^ cells) per well in a 96-well U-bottom microtiter plate in AIM-V in the absence of exogenous stimulus. For each condition we plated 4 replicate wells which were pooled at day 7 of analysis. Following 7 days of AP, cell supernatants were collected for downstream cytokine analysis and cells were analyzed by flow cytometry by staining for live cells (Live/DeadYellow), CD4, CD19, HLA-DR, OX-40 and 4-1BB.

#### Flow cytometry

After 7 days of AP or conventional B- or T cell reactions, CFSE-labeled cells were collected and pooled from replicate wells, washed with PBS, Fc-blocked with human IgG (Sigma-Aldrich, not used in the case of human IgG staining) and labeled with Live/Dead Aqua (Thermo Fisher Scientific) at 4°C. After washing with cold PBS containing 2mM EDTA and 2% FCS, cells were directly stained for surface markers using the fluorochrome-conjugated antibodies (key resources table).

Intracellular cytokine staining was performed on CFSE-labeled PBMCs of RRMS (REM) patients after adding GolgiStop protein transport inhibitor (BD Biosciences) at day 7 of AP culture. After 5 h in the presence of GolgiStop, CFSE-labeled PBMCs were pooled, Fc-blocked with human IgG and labeled with Live/Dead Aqua at 4°C and subsequently stained for CD19 on ice (key resources table). Following fixation and permeabilization with Cytofix/Cytoperm (BD Biosciences), cells were stained with cytokine-specific antibodies (key resources table) in PBS containing saponin (Applichem) and bovine serum albumin (BSA; Roth).

For the staining of nuclear transcription factors, CFSE-labeled PBMCs were pooled after 7 days of AP, Fc-blocked with human IgG and labeled with Live/Dead Aqua at 4°C and subsequently stained for CD19 on ice (key resources table). Upon stimulation, fixation and permeabilization with Foxp3/Transcription Factor Staining Buffer Set (eBioscience), cells were stained with specific antibodies to AID and T-bet (key resources table) or appropriate isotype control in Perm Wash solution (eBioscience).

Measurement of phosphorylated signaling molecules was performed on CFSE-labeled PBMCs of RRMS (REM) patients after 7 days of AP. After pooling replicate wells, cells were labeled with Live/Dead Aqua and stained with an antibody against CD19 on ice to prevent activation by the antibody. All centrifugation steps as well as buffers were pre-chilled at 4°C. Following wash steps in ice-cold PBS containing 2mM EDTA and 2% FCS, cells were fixed with 2% PFA for 15 min and after centrifugation permeabilized with 90% ice-cold methanol. Finally, the cells were stained with antibodies against phosphorylated epitopes of STAT1, STAT4 and STAT5 (key resources table) in PBS containing saponin and BSA. Measurements were performed on an LSR Fortessa Flow Cytometer (BD Biosciences), and data were analyzed with FlowJo (Tree Star). For the analysis, gates were set at first on singlets and live cells.

#### LegendScreen

CFSE-labeled PBMCs (approx. 8 × 10^7^ cells per donor) were prepared from 4 RRMS (NAT) patients were seeded as replicate wells with 2 × 10^5^ PBMCs/200 μL per well in AIM-V of 96-well U-bottom plates in order to allow AP, as described above. Following 7 days of AP, replicate wells of each donor were pooled, washed and B cells were separated using magnetic CD19 microbeads (Miltenyi) according to the manufacturer's instructions and checked for highest purity (>95%) with a CD20 antibody in flow cytometry. Isolated B cells were Fc-blocked with human IgG and labeled with Live/Dead Aqua at 4°C and B cell pools from each donor were subsequently prestained at 4°C with a donor-specific antibody mix containing antibodies to CD45 to allow multiplexing as well as antibodies to CD19-A700, CD27-PE-Cy7 and CXCR3-BV421 for further phenotyping. One donor did not have a CD45 antibody whereas the other donors included following antibodies CD45-PerCp-Cy5.5, CD45-APC or CD45-APC-Cy7 (key resources table). After staining and washing, labeled B cells from all donors were pooled (total 10 × 10^6^ cells), resuspended in cell staining buffer and kept on ice. The labeled B cell pool was then stained using the LegendScreen Human Cell Screening Kits (Biolegend), according to the manufacturer’s protocol with the following modifications. In brief, from the 332 PE-labeled antibodies targeting various surface antigens from the LegendScreen panel we pre-selected 165 of those antibodies based on the present RNA expression level of the respective antigens in CFSEhi and CFSEdim B cells using RNAseq data results (PRJEB23143). The resuspended labeled B cell pools were equally distributed and labeled with a 1:2 dilution of each of the pre-selected antibodies targeting 165 surface antigens, 7 isotype controls and a fluorescence minus one (FMO) control without PE-labeled antibody to control for specificity of surface antigen staining. Following 30 min incubation at 4°C and two washes, cells were resuspended in cell staining buffer (not fixed) and acquired for each sample 5-10 × 10^4^ B cells (>500 CFSEdim) on an LSR Fortessa Flow Cytometer (BD Biosciences). Data were analyzed with FlowJo (Tree Star). For the analysis, gates were set at first on singlets and live B cells, then CD45-based multiplexing of donors was deconvoluted and gates set on proliferating (CFSEdim) or resting (CFSEhi) CD27^+^ or CXCR3+ B cells. Median fluorescence intensity (MFI) of the PE signal was analyzed for each of the markers and controls screened.

As first part of the analysis, the MFI background of the respective isotype controls was subtracted from the MFI of the corresponding cell surface marker staining. In order to focus the analysis only on clearly expressed markers and those which show changes in expression between CD27^+^ CFSEdim and CFSEhi, following cut-offs were defined: a) MFI ratio of CD27+CFSEdim/CD27+CFSEhi to be at least >1.5 for upregulated markers and an MFI ratio of <0.5 for downregulated markers in the CD27+CFSEdim cell population. b) MFI to be > 250 in CD27+CFSEdim when respective CFSEdim/CSFEhi MFI ratio is > 1.5 and MFI to be > 250 in CD27+CFSEhi when respective CFSEdim/CSFEhi MFI ratio is < 0.5. Based on these cut-offs 68 markers were identified and categorized in markers with high cell surface expression (>7500 MFI), medium cell surface expression (1500–7500 MFI) and low cell surface expression (<1500 MFI). The data were accordingly plotted on heat maps using GraphPad Prism.

#### Iterative indirect immunofluorescence imaging (4i) procedure

Isolated frozen PBMCs of MS patients were thawed, washed in AIM-V medium and seeded in 96-well flat bottom microclear plates (key resources table). 100k PBMCs were cultured in 50μL AIM-V medium per well without any stimulus for 7 days at 37°C, 5% CO_2_. For each donor and condition, several replicate wells were seeded. The BTK inhibitor ibrutinib (key resources table) was used in a concentration of 8 nM and was compared with a DMSO vehicle control. As conventional B cell reaction, we stimulated the PBMCs with an anti-human IgM antibody (key resources table).

After 7 days, cells were fixed with 4% of paraformaldehyde (EMS) in PBS using a modified fixation protocol including an embedding step to immobilize non-adherent cells.^111^ After fixation, cells were subjected to sequential cycles of 4i procedure to allow for multiplexed antibody staining on the same sample, as previously described.^28^ Each 4i cycle included an elution, blocking, primary and secondary antibody incubation and an imaging step. Elution was carried at pH 2.5 using a buffer solution made of 0.5M Glycin (Sigma Aldrich), 1.2M Urea (Sigma Aldrich), 3M Guanidinium hydrochloride (Sigma Aldrich), 70mM TCEP-HCl (Thermo Fisher Scientific, Catalog # 20490 or Lucerna Chem, Catalog # UBP-P1020) in ddH2O. Addition of TCEP was required for the reduction of disulfide bonds. Three cycles of elution of 10 min each were performed to ensure complete removal of the bound antibodies. Blocking was carried out for 1h to prevent unspecific antibody binding using a solution of 2% BSA (Sigma Aldrich), 200 mM NH4Cl in PBS, in presence of 300mM Maleimide (Sigma Aldrich) to block free thiol groups present in cysteines. Blocking was followed by 1h incubation of primary and secondary antibody diluted in blocking buffer. Cell imaging was carried out with an imaging buffer (pH 7.4) made of 0.2M HEPES (Gibco) in ddH2O, in presence of 700mM N-Acetyl Cysteine (Sigma Aldrich) as antioxidant agent. Multiple washes (3x) in water or PBS were performed in between the different steps. Confocal microscopy images were acquired with the IN Cell Analyzer 6000 (GE Healthcare Life Sciences) automated microscope using the following setup: Nikon objective: 40× (0.95 NA, Plan Apo, Correction Collar 0.11–0.23, CFI/Lambda), 20 sections, Z-height of 0.5 μm, maximum intensity projection (MIP), 49 or 36 sites, channels: UV (406 nm), green (488 nm) and red (568 nm). Cy5 (625mn) channel was included in the last cycle to acquire Alexa Fluor 647 NHS Ester (Succinimidyl Ester) (Thermo Fisher Scientific) for cell segmentation. The image registration between consecutive cycles was preliminarily performed at the microscope level and later, computationally, at the image analysis stage.

##### Multiplexed image analysis

Image analysis was carried out using a built-in-house computational platform, named TissueMAPs (TMs) (developed in Pelkmans laboratory). Microscope images were registered in TMs and corrected for illumination artifacts; to allow for multiplexed phenotypic feature extraction at single cell level, images of consecutive 4i cycles were aligned and linked using DAPI nuclear staining and employing DAPI channel of the first cycle as reference. Tiled pyramids were then created to generate the map viewer, a zoomable multi-scale iterative object visualization. Nuclear and cellular segmentations were performed externally using the deep-learning-based segmentation algorithm cellpose.^88^ Cellpose-derived object masks from single images were generated in a loop using a customized Python script and then implemented in TMs as an additional channel. Visually-assisted supervised cell classification of B and T cell subtypes was performed using the random forests algorithm.

##### Nearest neighbor statistical analysis

The statistical hypothesis G test function was applied for the spatial density analysis of point patterns (cellular or nuclear centroid coordinates) to detect deviations from complete spatial randomness (CSR) in favor of clustering or dispersed patterns. In G function, the nearest neighbor distances (*r*) of each event to its closest event are computed and the cumulative distribution of distances is constructed. To mimic the CSR, random simulation is created with a random pattern using the same number of points within the same bounding box as in the experimental data and the G function is iteratively computed for each random event. The lowest and the highest G(r) values define a simulation or randomization envelope, which describes the variability of the G function under spatial randomness. G(r) values above the simulation envelope indicate clustering, as they are associated with a higher number of small inter-event distances (the cumulative distribution is steeper). Contrarily, G(r) values below the simulation envelope indicate dispersion or inhibition. Additional figure explanation: Dashed red line = theoretical cumulative distribution of distances; gray = variability of the G function; black line = observed cumulative distribution of distances. The G function was computed using the Spatstat R package.^89^ Additional explanations can be found here: https://darrylmcleod.com/wp-content/uploads/2016/06/Analysing-spatial-point-patterns-in-R.pdf.

#### Cytokine measurement

Supernatants were collected from the wells of cultured CFSE-labeled PBMCs or B-T cell cultures after 7 days (unstimulated). Cytokines were measured with Human T Helper Cytokine Panel LEGENDplex bead-based immunoassay (Biolegend; incl. measurement of 13 human cytokines: IL-2, 4, 5, 6, 9, 10, 13, 17A, 17F, 21, 22, IFN-γ and TNF) using flow cytometry according to the manufacturer's instructions. CXCL9 ELISA (R&D) was performed according to the manufacturer's instructions. Heat maps for cytokine responses were generated using GraphPad Prism.

#### RNA sequencing

RNA sequencing data are derived from previous analyses and was performed as previously described.^3^ In brief, 10.000–30.000 non-proliferating (CFSE^hi^) and AP (CFSE^dim^) CD19^+^ B cells and CD3^+^CD4^+^ T cells from 5 to 6 RRMS patients (REM; nihil; *n* = 5 for T cells and *n* = 6 for B cells) were sorted following 7 days of unstimulated *in vitro* culture and labeling with Live/Dead Aqua and antibodies against CD3, CD4, CD8 and CD19 (key resources table) using a FACSAria III (BD Biosciences). Sorted cells were adjusted to the same cell number for CFSE^hi^ and CFSE^dim^ and RNA isolated with the PicoPure RNA Isolation kit (Thermo Fisher Scientific) after phenol (Qiazol)/chloroform extraction. RNA sequencing was carried out on the Illumina HiSeq 2500 with single read (1 × 125bp) approach using the TruSeq SBS Kit v4-HS (Illumina) at the Functional Genomics Center Zurich (FGCZ) as previously described.^3^ Following data cleaning, differential gene expression analysis between CFSE^dim^ and CFSE^hi^ cell samples was performed using the R/bioconductor package edgeR in which the normalization factor was calculated by trimmed mean of M values (TMM) method.^112^ P-values were adjusted for multiple testing using the Benjamini-Hochberg procedure. Genes not present (<10 counts per gene) in at least 50% of samples from one condition were discarded from further analyses. For cluster analysis, a *p*-value <0.01 was applied. Thresholds log2 fold change >0.5 and adjusted *p*-values <0.01 were used for pathway analysis with Ingenuity (Qiagen). A gene list involved in germinal center-like formation was generated based on reported signature genes in literature.^113^^,^^114^^,^^115^ Significance for enrichment in expression of those genes in AP B cells within these pathways was calculated based on a Fishers exact test.

#### Single cell RNA and VDJ sequencing

Non-proliferating (CFSE^hi^) and AP+ (CFSE^dim^) CD19^+^ B cells from 5 HDs, 6 RRMS (REM; nihil) and 5 natalizumab-treated RRMS (NAT) patients were sorted following 7 days of unstimulated *in vitro* PBMC culture (Autoproliferation). One CFSE^hi^ sample from a NAT donor had to be excluded during the procedure due to loss in cell amount and cell quality, which finally resulted in a total of 31 B cell samples from above mentioned donors.

In brief, CFSE-labeled cells were stained upon culture with Live/DeadYellow and antibodies against CD3, CD4, CD8 and CD19 (key resources table) using a Sony SH800 cell sorter. The donors were selected to be on group comparison age- and gender matched. We collected a maximum of 1 × 10^4^ cells and all cell populations were rechecked for at least 95% purity in flow cytometry. The patient demographics are summarized in Table S1. Sorted cells were collected in PBS containing 10% FCS, immediately put on ice following sorting and centrifuged in RNase-free Tubes for 10 min at 400xg. The cell pellet was resuspended in 40ul of PBS containing 10% FCS and loaded into the 10x Genomics Chromium Controller for droplet-encapsulation. cDNA libraries were prepared using Chromium Next GEM Single Cell 5′ Library & Gel Bead Kit v1.1 (10x Genomics) and Chromium Single Cell 5′ Library Construction Kit (10x Genomics) according to the manufacturer’s instructions. BCR-enriched libraries were prepared for each sample using Chromium Single Cell V(D)J Enrichment Kit (Human B Cell). All libraries were sequenced using NovaSeq 6000 (Illumina) and NovaSeq 6000 S2 Reagent Kit v1.5 (300 cycles) (Illumina) to get a sequencing depth of 50K reads/cell (whole transcriptome libraries) or 10K reads/cell (BCR enriched libraries).

##### Single-cell RNAseq data processing and quality control

The Alevin/Salmon suite was used to align, quantify, collapse, and whitelist cellular barcodes on 31 samples (Chromium Next GEM Single Cell 5′ Libraries) based on the GRCh38 reference genome (v33 GENCODE/Ensembl annotation). For each sample, Alevin targeted for 5000 cells and <20% noisy cellular barcodes (i.e. cells beyond the ‘knee’-estimate on the cumulative frequency of observed barcodes). In all samples, cellular barcodes with a 1-ball levenshtein distance were collapsed. The non-filtered gene-by-count matrices were filtered for empties (i.e., barcodes containing ambient RNA) using EmptyDrops.^116^ In the advent of co-capture of multiple cells in the same physical droplet, such 'doublets/multiplets’ were removed using *scDblFinder*.^117^ Contrary to convention, doublet removal was deliberately conducted before any other quality control (QC). With regards to the QC, we did not employ global thresholds for QCing the cells. Instead for each gene-by-count matrix (GEX matrix), three quality control metrics were computed per cell: (a) total UMI count (b) number of detected genes (detection implying >1 UMI for said gene) (c) proportion of mitochondrial reads. Cells lying >3 median-absolute-deviation (MAD) away, with respect to the three aforementioned metrics, were removed. Genes with zero-expression in all samples were also removed.

##### B cell numbers post-QC per donor

HD1 (resting: 3805, AP: 2960), HD2 (resting: 3915, AP: 3566), HD3 (resting: 891, AP: 3011), HD4 (resting: 3118, AP: 4503), HD5 (resting: 3277, AP: 3310), REM1 (resting: 3027, AP: 3269), REM2 (resting: 2536, AP: 2387), REM3 (resting: 2380, AP: 2928), REM4 (resting: 2510, AP: 2308), REM5 (resting: 3297, AP: 3121), REM6 (resting: 3845, AP: 3281), NAT1 (resting: 3072, AP: 4958), NAT2 (resting: 3392, AP: 4045), NAT3 (resting: 3451, AP: 3023), NAT4 (resting: 3172, AP: 4242) and NAT5 (resting: 0, AP: 3078).

##### Normalization and dimensionality reduction

For each GEX matrix, library size factors were determined per cell and used to compute (log-)normalized counts.^118^ We adjusted for batch effects (i.e., differences in sequencing depth between samples) by further (re)scaling the size factors as described in *batchelor.*^119^ The downstream analysis and visualizations use these (log-)normalized counts (unless stated otherwise). We also used SCTransform to variance stabilize the counts, identify highly variable genes, and scale the data.^105^ The ‘top’ 100 principal components were computed using the 3000 most highly variable genes; mitochondrial genes were deemed a nuisance source of variation and thus their percentage abundance was ‘regressed out’ (using the `vars.to.regress` parameter in SeuratSCTransform()).

##### Integration and unsupervised clustering

The GEX matrices from SCTransform were used for integration and unsupervised clustering. To avoid clustering on the clonality, V-D-J genes were omitted solely for the purpose of clustering (we retained these genes for other analyses). Conos was used for integration and clustering of the 31 CD19^+^ samples.^120^ Conos allows identification of transcriptionally similar cells across the datasets while accounting for batch effects. With regards to unsupervised clustering, Conos was parameterized to use the Leiden clustering algorithm. Several Leiden ‘resolutions’ were evaluated, until finally settling at resolution 1. This resulted in 9 clusters, but the smallest cluster (∼2300 cells) was filtered out due to high percentage of mitochondrial content (>25% mean mitochondrial genes). For visualization purposes, we used the Uniform Manifold Approximation and Projection (UMAP) algorithm to further dimensionally reduce the integrated dataset to 2-dimensions (Figure 6A).

##### Annotating clusters

For a transcriptional overview of cell type expression (Figure 6B), we manually curated a list of known cell subpopulation and other key markers using PanglaoDB database,^121^ from literature^122^ and overexpressed AP+ B cell surface markers from Legendscreen phenotyping. The signature markers for the 8 clusters (Figure S5C) were determined using *scran::findMarkers* using the *test.type = "wilcox"* and *test.type = "binom"*. Other parameters of *findMarkers()* were *pval.type = "some"*, *min.prop = 0.6*, and we blocked on the samples. Considering the 8 clusters, we chose not to be strict (*pval.type = ”all”*) or lax (i.e., *pval.type = ”any”*). For both tests, the FDR threshold for a putative signature gene was fixed at 0.01%, but we evaluated various logFC and AUC thresholds. This was done primarily to limit the sheer number of signature genes that two clusters (‘MBC_3’ and ‘PB_2’) would otherwise yield. Therefore, a threshold of logFC >2 and AUC >0.65 was applied on all clusters; except MBC_3 and PB_2, where we used a logFC>5.

##### Differential gene expression analysis

For all DGE analysis, we aggregated our data (‘psuedobulk’ed) using *scuttle* (*aggregateAcrossCells()*/*aggregateAcrossGroups()*) before performing the differential gene analysis using *limma-voom*.^91^^,^^118^^,^^123^ Aggregation was done on the sum of counts. The DEG analysis itself was done using the *muscat* package.^124^ The Molecular Signatures Database (MSigDB) was used to identify 179 IFN-γ response pathway genes (HALLMARK_INTERFERON_GAMMA_RESPONSE); pathway genes with zero-expression were omitted. To study the IFN-γ pathway in the CXCR3+ subset of cells within each celltype, we (1) identify and extract the CXCR3+ cell subpopulation from all samples, (2) annotate all samples as either AP+ or resting; AP+ and resting are the two groups that represent the comparison of interest (3) aggregate the gene-expression (‘pseudobulk’) by cell-type and sample (aggregation was done on the sum of counts), (4) for each of the 8 cell types, we fit a linear model for each gene. That is, for each cell type we compare the gene-expression in the ‘AP’ samples vs. the ‘Resting’ samples. In order to identify exclusive DEGs in CXCR3+ AP+ B cells of MS patients, we limited our analysis on to only the AP samples and then split our data into two subpopulations of CXCR3+ and CXCR3-cells. We fit models with 3 or 4 conditions (HD, REM, NAT, and MS; the ‘MS’ label is assigned to cells that are either ‘REM’ or ‘NAT’). Thus, our DEG analysis has the contrasts for HDvREM, HDvNAT and HDvMS. DEGs were identified in both subpopulations (FDR <0.01 and logFC >1), but Figure 6G only shows genes that were exclusively differentially expressed in the CXCR3+ B cell subpopulation (i.e., and are not DE in the CXCR3-subpopulation). The aforementioned DEG analysis was repeated for each cell type (cluster) (Figure S5H).

##### VDJ sequencing data processing and quality control

The VDJ sequence data from 31 samples was processed using the Cellranger VDJ pipeline (v6) for full-length VDJ sequence reconstruction and paired (Heavy/Light) chain calling. Sequence contigs and scaffolds were assembled using the 10X VDJ reference (GRCh38-alts-ensembl-6). Only barcodes having exactly one Heavy and Light chain were retained. Barcodes missing a corresponding gene-expression vector were also retained. MiXCR^125^ and Platypus v3^126^ were used to extract the VDJ region information, germline sequences and various exploratory tasks. Clonotyping was performed using V-J gene identity and 70% CDR3 nucleotide sequence homology. Specifically, for two or more barcodes to be grouped into the same clonotype, they need to share the same V and J gene, as well as have >70% CDR3 NT-sequence overlapping in their heavy chain. We adopt the IMGT definition of CDR3 that excludes the CYS and TRP/PHE regions of the junction. For each expanded clonotype, we examined the distribution of cell types and isotypes found within. In Figure 6C, the values within the boxes represent the number of clonotypes that have at least one clonal cell of both cell types.

#### Processing and analysis of single nuclei RNAseq data

Nuclei were isolated from several fresh-frozen 10μm sections of well characterized MS brain tissue blocks with highly inflamed leptomeninges and adjacent gray matter from three MS donors (MMS1: male, 40years, SPMS; MMS2: female, 44years, SPMS; MMS3: female, 53years, SPMS). Given the small number of donors and to ensure that extracted data are reproducible, we isolated different sections from the same brain tissue twice per donor (sample A and B) to obtain and run 2 biological replicate samples per donor and brain tissue block. Single nuclei have been isolated and processed for snRNAseq as previously described.^127^ Isolated nuclei were loaded on the 10x Single Cell Next GEM G Chip. cDNA libraries have been prepared using the Chromium Single Cell 3′ Library and Gel Bead v3.3 kit according to the manufacturer’s instructions. cDNA libraries were sequenced using Illumina NovaSeq 6000 System and NovaSeq 6000 S2 Reagent Kit v1.5 (100 cycles) in order to obtain a sequencing depth of minimum 30K reads/nucleus. All samples were processed with simpleaf (v0.15.1),^128^ using the GRCh38 reference human genome and the ensembl Homo_sapiens GRCh38.96 reference annotation. Ambient RNA was removed using Cellbender (v0.3.0).^129^ We identified putative doublets using scDblFinder (version 1.4.0), applied to each sample separately (multiSampleMode = “split”), with all other parameters default. After removing doublets, we removed poor quality cells by excluding cells with fewer than 300 transcripts or fewer than 100 features, percentage of exonic reads lower than 10% or percentage of exonic reads higher than 75%. This resulted in 24.939 nuclei across 6 samples passing QC. We identified 2000 highly variable genes using the FindVariableFeatures function in Seurat (v5.0.1), calculated 50 principal components, and used these as input to Harmony (v1.2.0), with parameter theta set to 0.1 and other parameters set to default. To identify clusters, we used the FindClusters function in Seurat applied to the Harmony outputs, with resolution set to 0.1.

#### Paired methylome and transcriptome library construction and analysis

Non-proliferating (CFSE^hi^) and AP+ (CFSE^dim^) CD19^+^ CD27^+^ B cells from 6 natalizumab-treated female RRMS (NAT) patients were sorted following 7 days of unstimulated *in vitro* PBMC culture (AP). CFSE-labeled cells were stained upon culture with Live/DeadViolet and antibodies against CD3, CD19 and CD27 (key resources table) using a Sony SH800 cell sorter. We collected a maximum of 2 × 10^4^ cells and all cell populations were rechecked for at least 95% purity in flow cytometry. The patient demographics are summarized in Table S1. Sorted cells were collected in PBS containing 10%FCS, immediately put on ice following sorting and centrifuged twice with cold PBS in RNase-free Tubes for 10 min at 400xg. The cell pellets were stored at −80°C until downstream methylome and transcriptome library construction.

Methylome libraries for 12 samples (AP+ and resting memory B cell samples from six individuals) were constructed by applying the post-bisulfite adaptor tagging (PBAT) method on lysed cells as previously described^130^ with 10–12 PCR cycles. Sequencing was conducted on Illumina NovaSeq 6000 (2 × 150bp). Trim Galore was used to clip reads in both 5′ and 3′ ends of R1 and R2 for 9bp with parameters --clip_r1 9, --three_prime_clip_R1 9, --clip_r2 9, --three_prime_clip_R2 9, trim adapters and remove low-quality reads by default parameters. Clean reads were subsequently aligned to Homo Sapiens GRCh38 using Bismark with PBAT mapping strategies for paired end data. Reads were first mapped in paired end mode, aligned reads were then deduplicated and methylation extracted, unmapped R1 was then mapped in single-end mode (--pbat), deduplicated and methylation extracted. Unmapped R2 was also mapped in single-end mode, deduplicated and methylation extracted. All CpG calls were then combined into a single bedgraph with bismark2bedGraph and merged reads mapped to both strands of CpG dinucleotide by coverage2cytosine with --merge_CpG parameter. CpGs with minimum 10X coverage in 10 out of 12 samples were used for differential methylation position (DMP) analysis, which was done using Limma, including proliferation status and age in the model and considering the paired design. Methylation level of CpGs with less than 10X coverage was replaced by NA. A minimum methylation difference of 10% and *p*-value <0.001 were used as selection criteria for candidate DMPs. CpGs were annotated by genes and features downloaded in Ensembl with genes extended upstream by 2kb.

Transcriptome libraries were constructed from the same samples, except for one sample pair, which was excluded due to limited input material. SMARTer Total Stranded RNA-seq library construction with ribosomal depletion was performed following manufacturer’s instructions. Samples were sequenced on NovaSeq S4 lane (2 × 150bp). QC and alignment were performed using nf-core/rnaseq with GRCh38 as reference and annotation files were from Igenomes NCBI. Additional to the typical pipeline, 3bp from 5′ R1 and 3′ of R2 were trimmed. Genes with less than 10 counts in all samples were excluded. Differential analysis and normalization were done by DESeq2 considering paired sample design. A 1.2 foldchange (log2) and adj. *p*-value <0.01 were used as threshold for DEG detection. Pathway enrichment analysis was performed using Gene Set Enrichment Analysis (GSEA).

### Quantification and statistical analysis

Statistical analyses were performed with GraphPad Prism. Multiple comparison analyses were assessed with a non-parametric one-way Kruskal-Wallis test and Dunn’s multiple comparisons test. Non-parametric two-tailed Mann-Whitney *U* test was performed on data with only two groups. Correlation was calculated using a 2-tailed Pearson’s correlation test. *p* values are reported in the figures and figure legends where significant. Error bars are shown as standard error of the mean (SEM) and are indicated in the figures. Sample sizes were chosen based on previous experimental data.^3^ Other quantitative and statistical methods are noted above according to their respective technologies and analytic approaches (e.g., scRNAseq).

## Supplemental information

Document S1. Figures S1–S7 and Tables S1 and S2

Table S3. Signature genes of each B cell cluster based on scRNA-seq data, related to Figures 6 and S5, and Table S1

Table S4. Maturation within AP+ CXCR3+ B cell clonotypes Expanded clonotypes, where each clonotype is matured (i.e., hypermutated), contains a mix of cell types and mix of isotypes (Sheet 1), and demonstrates differentiation from naive and/or memory to plasmablasts (Sheet 2), related to Figures 6 and S6, and Table S1.

Table S5. Signature genes expressed in identified cell clusters in snRNA-seq of inflamed meningeal MS tissues, related to Figures 7 and S7

Document S2. Article plus supplemental information
