# Supplementary material for: T-bet+ CXCR3+ B cells drive hyperreactive B-T cell interactions in multiple sclerosis
Source: Cell Rep Med. 2025 Mar 18;6(3):102027. doi: 10.1016/j.xcrm.2025.102027 (PMC11970401; doi:10.1016/j.xcrm.2025.102027)
Supplement: Document S1. Figures S1–S7 and Tables S1 and S2 [file mmc1.pdf]

Cell Reports Medicine, Volume *6*

Supplemental information

T-bet+ CXCR3+ B cells drive hyperreactive

B-T cell interactions in multiple sclerosis

Ivan Jelcic, Reza Naghavian, Imran Fanaswala, Will Macnair, Cinzia Esposito, Daniela Calini, Yanan Han, Zoe Marti, Catarina Raposo, Jacobo Sarabia del Castillo, Pietro Oldrati, Daniel Erny, Veronika Kana, Galina Zheleznyakova, Faiez Al Nimer, Björn Tackenberg, Ina Reichen, Mohsen Khademi, Fredrik Piehl, Mark D. Robinson, Ilijas Jelcic, Mireia Sospedra, Lucas Pelkmans, Dheeraj Malhotra, Richard Reynolds, Maja Jagodic, and Roland Martin

FIGURE S1

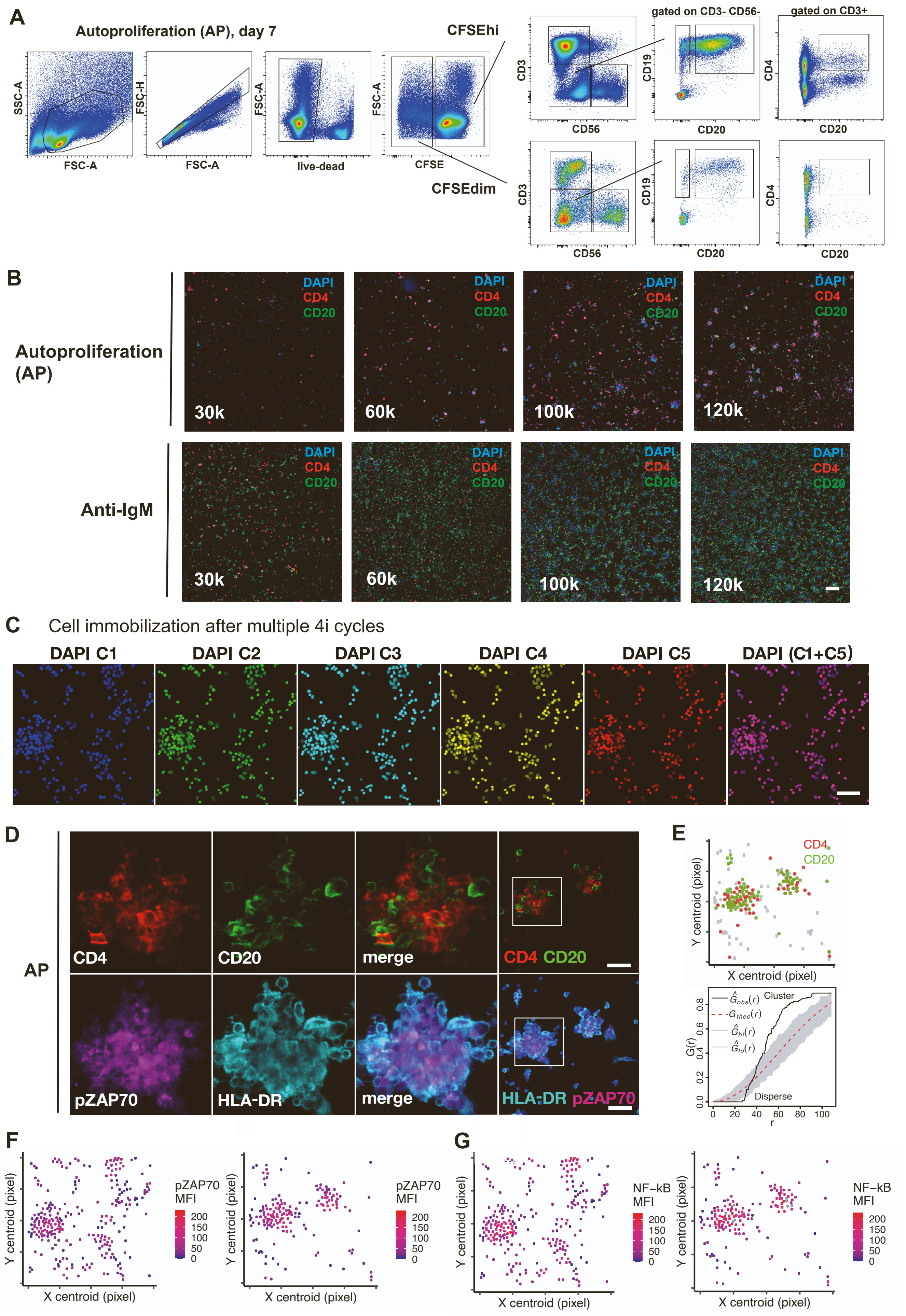
A
Autoproliferation (AP), day 7
CFSEhi
CFSEdim
SSC-A
FSC-A
FSC-H
FSC-A
FSC-A
live-dead
FSC-A
CFSE
CD3
CD56
gated on CD3- CD56-
CD19
CD20
gated on CD3+
CD4
CD20
B
Autoproliferation (AP)
Anti-IgM
DAPI
CD4
CD20
30k
60k
100k
120k
C
Cell immobilization after multiple 4i cycles
DAPI C1
DAPI C2
DAPI C3
DAPI C4
DAPI C5
DAPI (C1+C5)
D
AP
CD4
CD20
merge
CD4 CD20
pZAP70
HLA-DR
merge
HLA-DR pZAP70
E
CD4
CD20
Y centroid (pixel)
X centroid (pixel)
Cluster
Disperse
G(r)
r
F
pZAP70 MFI
200
150
100
50
0
Y centroid (pixel)
X centroid (pixel)
G
NF−kB MFI

Figure S1. 4i procedure allows spatial and multi-parametric visualization of AP-associated BTECs. Related to Figure 1 and Table S1.

(**A**) Expression of CD19 and CD20 among B cells during AP (n = 3 RRMS, NAT).

(**B**) IF images (well-view, 49 stitched-sites) showing B cells marked by CD20 (green), T cells marked by CD4 (red) and nuclear staining by DAPI (blue) in AP (top panel) and IgM conditions (bottom panel). Images from left to right show increasing cell density as indicated. Scale bar: 200 µm.

(**C**) IF images depicting nuclear DAPI staining of five consecutive 4i cycles. Image at the right end shows the superimposition of DAPI intensity signals between the first and the last 4i cycle, indicative of robust immobilization of lymphocytes over multiple 4i cycles. Scale bar: 50 µm.

(**D**) Molecular characterization of B-T cell clusters using the 4i protocol (independent experiment). Multiplexed IF images of T cells marked by CD4 (red) and B cells marked by CD20 (green) (top panel) stained for pZAP70 (magenta) and HLA-DR (cyan). Images from left to right show higher magnification of white squares in 1-site view images at the right end. Scale bar: 50 µm. Representative image from one of the replicate wells in one of tested donor PBMCs (n = 3 RRMS, NAT).

(**E**) Top panel: 2D-projection of cell morphology centroids from experiment in S1D, colored by B cells (CD20, green) and T cells (CD4, red). Cell assignments were determined using random forest classification. Bottom panel: G(r) function computed on cell morphology centroids from experiment in S1D. G(r) values above or below the simulation envelope imply clustering or dispersion, respectively.

(**F, G**) 2D-projection of local morphology centroids from experiment in Figure 1F (left panel) and S1D (right panel), colored by the mean fluorescence intensity values of pZAP70 (F) or NF-kB (G) to visualize the spatial intensity distribution of T cell signaling and inflammatory signaling, respectively, in BTECs.

FIGURE S2

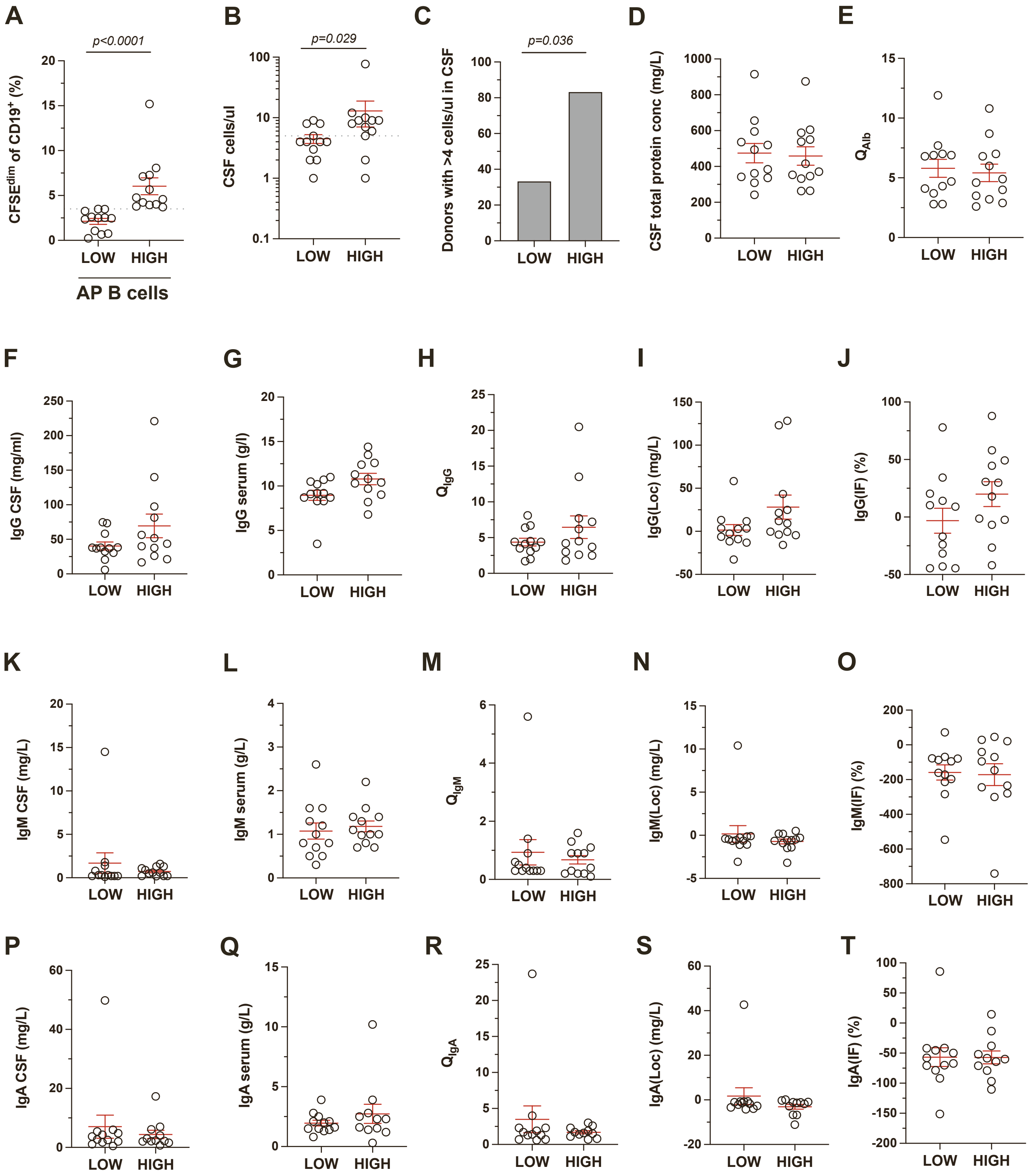
A
p<0.0001
CFSE^{dim} of CD19^{+} (%)
LOW HIGH
AP B cells
B
p=0.029
CSF cells/ul
LOW HIGH
C
p=0.036
Donors with >4 cells/ul in CSF
LOW HIGH
D
CSF total protein conc (mg/L)
LOW HIGH
E
Q_{Alb}
LOW HIGH
F
IgG CSF (mg/ml)
LOW HIGH
G
IgG serum (g/l)
LOW HIGH
H
Q_{IgG}
LOW HIGH
I
IgG(Loc) (mg/L)
LOW HIGH
J
IgG(IF) (%)
LOW HIGH
K
IgM CSF (mg/L)
LOW HIGH
L
IgM serum (g/L)
LOW HIGH
M
Q_{IgM}
LOW HIGH
N
IgM(Loc) (mg/L)
LOW HIGH
O
IgM(IF) (%)
LOW HIGH
P
IgA CSF (mg/L)
LOW HIGH
Q
IgA serum (g/L)
LOW HIGH
R
Q_{IgA}
LOW HIGH
S
IgA(Loc) (mg/L)
LOW HIGH
T
IgA(IF) (%)
LOW HIGH

Figure S2. Higher levels of peripheral AP+ B cells associate with increased CSF cell count but not with enhanced antibody responses in CSF. Related to Figure 2 and 5 and Table S1 and S2.
(**A**) RRMS (REM) patients were grouped in LOW (n = 12) and HIGH (n = 12) responders based on their levels of peripheral AP+ B cells (low < 3.6% and high > 3.6%) and from whom basic CSF parameters were available from clinical routine. The threshold of 3.6% AP+ B cells was defined as the median of all AP+ B cell frequency values for this patient cohort.
(B, C) Depicted is (B) the number of cells in CSF and (C) the relative proportion of donors with a cell count of >4 per µl CSF indicative of an active intrathecal inflammatory response, in the low and high AP+ B cell group, (LOW n = 12, HIGH n = 12; (B) Mann-Whitney *U* test, (C) Mann Fisher`s exact test).
(D, E) CSF total protein concentration and CSF/serum albumin quotient Q_{Alb} as indicators for blood-brain barrier integrity, separated by donors with low and high AP+ B cells.
(F-J) Total IgG in CSF and serum, as well as quotients and fractions of intrathecally produced IgG in donors with low and high AP+ B cell.
(K-O) Total IgM in CSF and serum, as well as quotients and fractions of intrathecally produced IgM in donors with low and high AP+ B cell.
(P-T) Total IgA in CSF and serum, as well as quotients and fractions of intrathecally produced IgA in donors with low and high AP+ B cell levels.

FIGURE S3

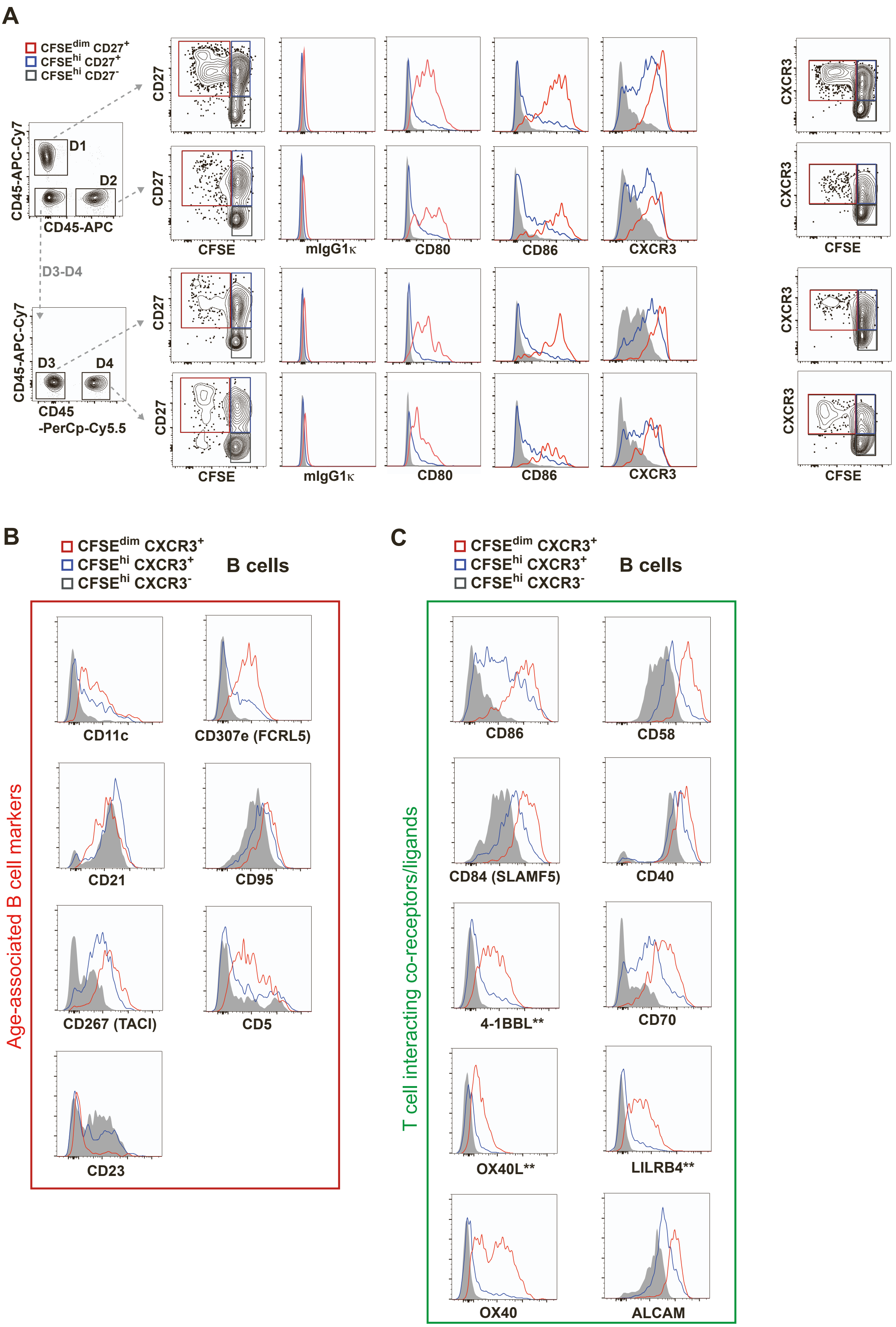
A
CFSE^{dim} CD27^{+}
CFSE^{hi} CD27^{+}
CFSE^{hi} CD27^{-}
CD45-APC-Cy7
D1
D2
CD45-APC
D3-D4
D3
D4
CD45
-PerCp-Cy5.5
CD27
CFSE
mIgG1κ
CD80
CD86
CXCR3
B
CFSE^{dim} CXCR3^{+}
CFSE^{hi} CXCR3^{+}
CFSE^{hi} CXCR3^{-}
B cells
Age-associated B cell markers
CD11c
CD307e (FCRL5)
CD21
CD95
CD267 (TACI)
CD5
CD23
C
T cell interacting co-receptors/ligands
CD58
CD84 (SLAMF5)
CD40
4-1BBL**
CD70
OX40L**
LILRB4**
OX40
ALCAM

Figure S3. AP B cells express characteristic markers of age-associated B cells. Related to Figure 3 and Table S1.

(**A)** Gating strategy for sample deconvolution based on CD45 barcoding, subgating on $CD27^{-}CFSE^{hi}$ naïve resting B cells (grey), $CD27+CFSE^{hi}$ memory resting B cells (blue) and $CD27+CFSE^{dim}$ memory AP+ B cells (red). Exemplary histogram plots of CD80, CD86 and CXCR3 expression and the isotype control in gated subpopulations of each donor. In addition, dot plots subgating on $CXCR3^{-}CFSE^{hi}$ (grey), $CXCR3+CFSE^{hi}$ B cells (blue) and $CXCR3+CFSE^{dim}$ AP+ B cells (red) are shown on the right.

(**B, C**) Representative histogram plots for expression of red and green highlighted key markers for age-associated B cells (B) and T cell interacting co-receptor/ligands (C) in $CXCR3\text{-}CFSE^{hi}$ naïve resting B cells (grey), $CXCR3+CFSE^{hi}$ memory resting B cells (blue) and $CXCR3+CFSE^{dim}$ memory AP+ B cells (red).

FIGURE S4

A

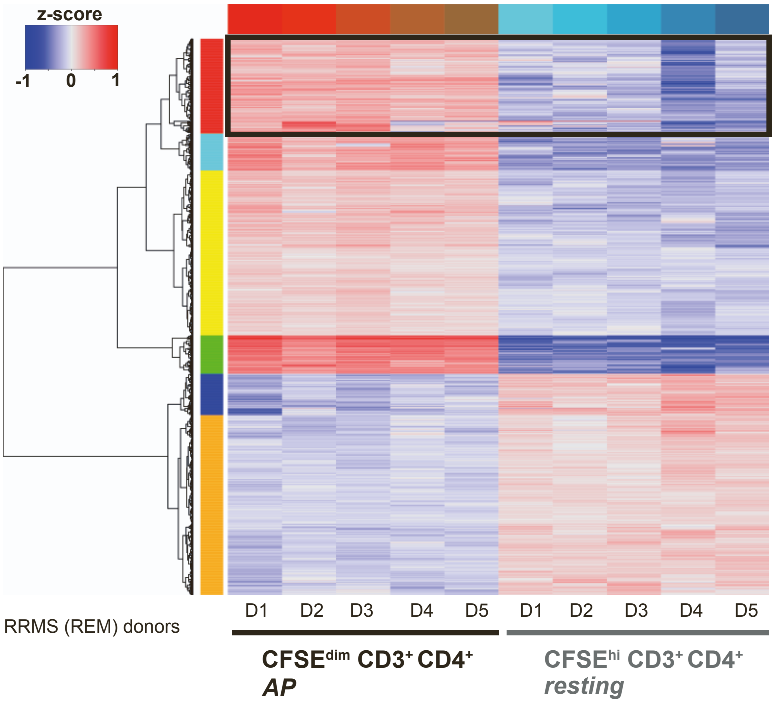

Term	ID	p
immune response	GO:0006955	8.37E-12
interferon-gamma-mediated signaling pathway	GO:0060333	1.73E-05
mitotic cell cycle	GO:0000278	6.88E-07
chemokine-mediated signaling pathway	GO:0070098	1.12E-06
**T cell costimulation**	**GO:0031295**	**3.23E-05**
glycolytic process	GO:0006096	8.41E-05

B

C

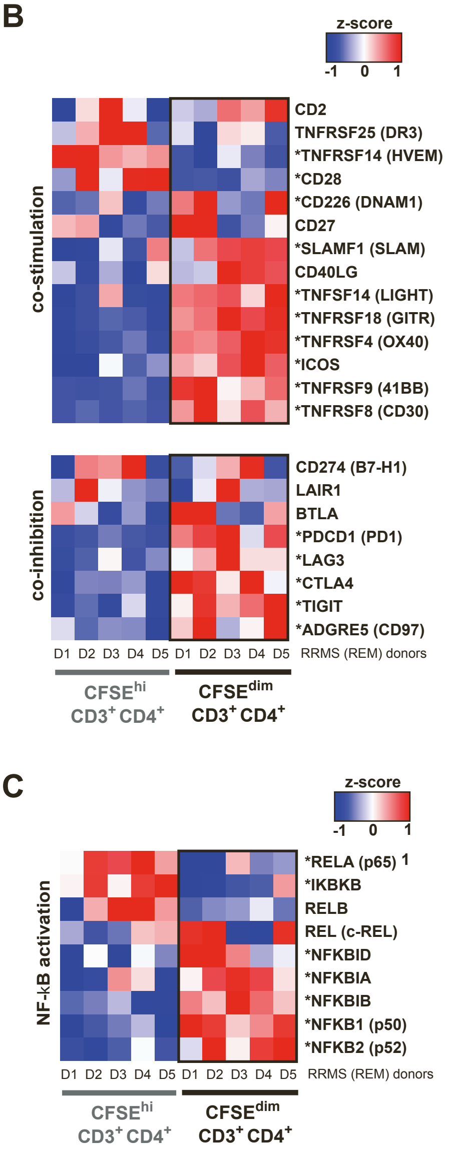

D

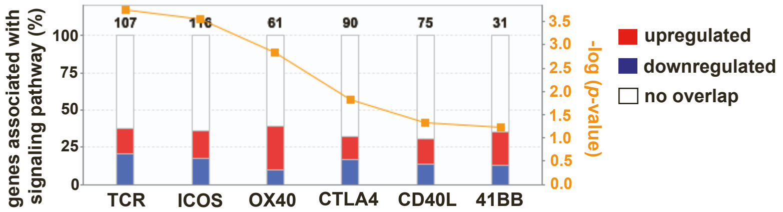

E

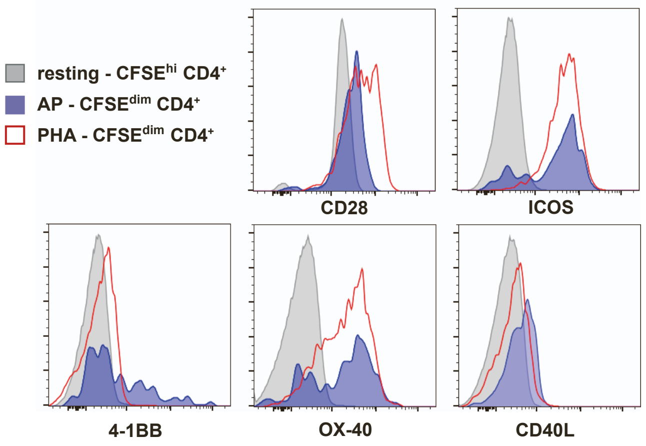

F

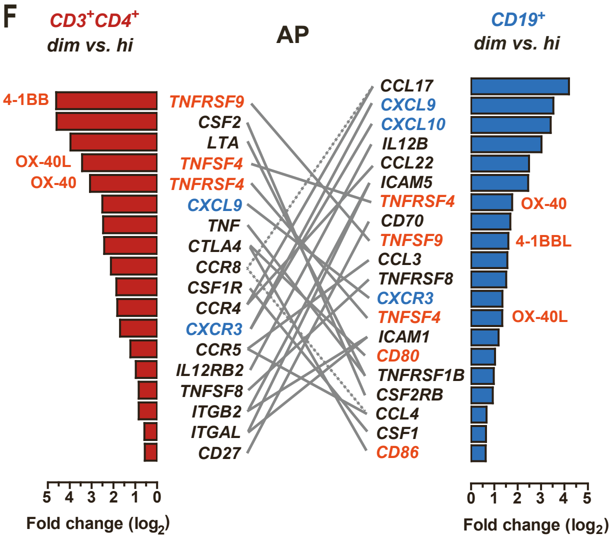

Figure S4. AP T helper cells reveal a defined co-receptor expression signature that overlap with upregulated cognate ligands on AP B cells. Related to Figure 4-6, Figure S3 and Table S1.
(**A**) Heat map of significant DEGs (log2 > 0.5; fdr < 0.01) in sorted $CFSE^{hi}$ and $CFSE^{dim}$ CD4+ T cells in RRMS patients (n = 5; RRMS (REM); nihil) using RNA sequencing. The differential expression is shown as z-score based on the RPKM values. Genes are categorized based on similar pathway annotations (left colored boxes). Red highlighted box includes DEGs within certain immune-related GO pathways as depicted in the bottom table.
(**B, C**) Co-receptors (B) and NF-κB pathway-related key genes (C) were analyzed for differential expression in sorted $CFSE^{hi}$ and $CFSE^{dim}$CD3+CD4+ cells based on transcriptome data. The differential expression is expressed by the z-score based on the RPKM values. Significant DEGs are indicated by asterisks and aliases for gene names depicted in brackets.
(**D**) The transcriptome data of $CFSE^{dim}$ over $CFSE^{hi}$ CD3+CD4+ T cells was analyzed for the TCR and most affected canonical co-receptor pathways. The bar charts indicate the number of DEGs (up- and downregulated) within the corresponding pathway. The pathways are grouped according to the magnitude of significant dysregulation (orange). A threshold of log2 fold change >0.5 and adjusted p-values <0.01 was used for pathway analysis with Ingenuity (Qiagen).
(**E**) Surface expression for dedicated co-receptors on resting (grey), AP+ (blue) and PHA-stimulated (red) CD4+ T cells upon 7 days of *in vitro* culture.
(**F**) Differential gene expression of co-receptor/-ligands in AP+ B cells (blue bars) and CD4+ T cells (red bars) depicted as fold change between $CFSE^{dim}$ and $CFSE^{hi}$ subsets (log2 > 0.5; fdr < 0.01). Cognate co-receptor and ligand interactions are connected with lines. The CXCR3-CXCL9/10 axis is highlighted in blue and the OX-40/OX-40L as well as 4-1BB/4-1BBL co-receptor pairs in red.

FIGURE S5

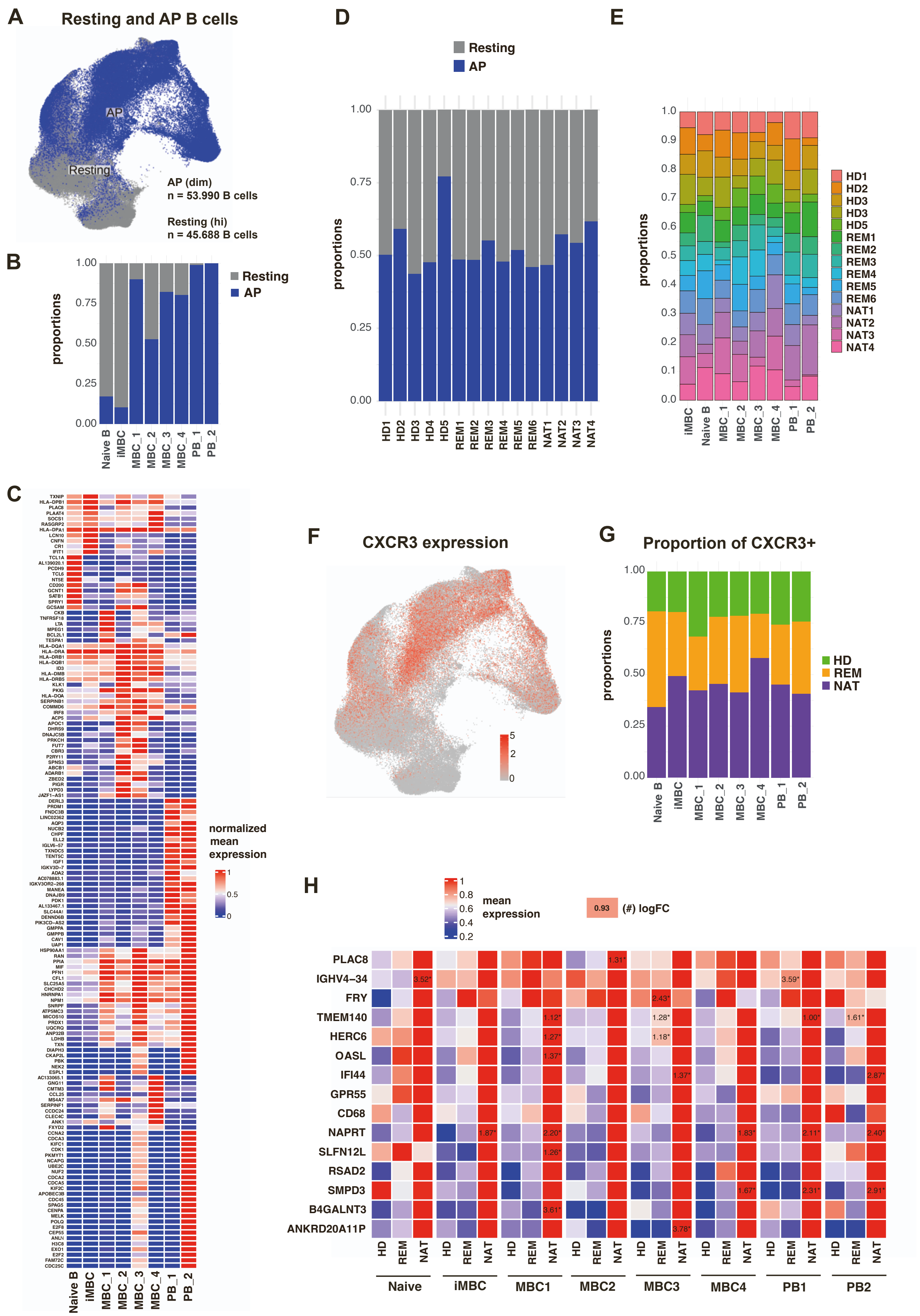
A
Resting and AP B cells
AP
Resting
AP (dim)
n = 53.990 B cells
Resting (hi)
n = 45.688 B cells
B
Resting
AP
proportions
Naive B
iMBC
MBC_1
MBC_2
MBC_3
MBC_4
PB_1
PB_2
C
normalized mean expression
D
Resting
AP
proportions
HD1
HD2
HD3
HD4
HD5
REM1
REM2
REM3
REM4
REM5
REM6
NAT1
NAT2
NAT3
NAT4
E
HD1
HD2
HD3
HD3
HD5
REM1
REM2
REM3
REM4
REM5
REM6
NAT1
NAT2
NAT3
NAT4
F
CXCR3 expression
G
Proportion of CXCR3+
HD
REM
NAT
H
mean expression
(#) logFC
PLAC8
IGHV4–34
FRY
TMEM140
HERC6
OASL
IFI44
GPR55
CD68
NAPRT
SLFN12L
RSAD2
SMPD3
B4GALNT3
ANKRD20A11P
Naive
iMBC
MBC1
MBC2
MBC3
MBC4
PB1
PB2

Figure S5. Signature genes, sample-to sample / donor-to-donor variability, and MS DEGs across B cell subsets. Related to Figure 6, Table S1 and Table S3.

(**A**) UMAP of 99.678 single cells from resting and AP+ B cell samples of HD (n = 5), REM (n = 6) and NAT (n = 5) donors. Each dot corresponds to a single cell (grey is resting and blue is AP).

(**B**) Per-cluster proportions of resting and AP+ B cells.

(**C**) Signature genes that have been identified for each B cell cluster.

(**D, E**) Donor-to-Donor variability with regard to (D) proportions of resting and AP+ B cells and (E) proportions of B cell subsets. One NAT patient is omitted because it is missing a resting/CFSEhi B cell sample.

(**F**) Representation of CXCR3-expressing B cells in UMAP plot. Each dot corresponds to a single cell, colored by a red gradient for CXCR3 expression.

(**G**) Proportion of CXCR3+ B cells within HD, REM and NAT donor groups and across the 8 identified B cell clusters.

(**H**) Distribution and expression of identified CXCR3+ and MS DEGs across the 8 identified B cell clusters gated on CXCR3+. Heatmap shows mean "pseudobulked" expression and numbers in boxes indicate logFC for significant expression differences.

FIGURE S6

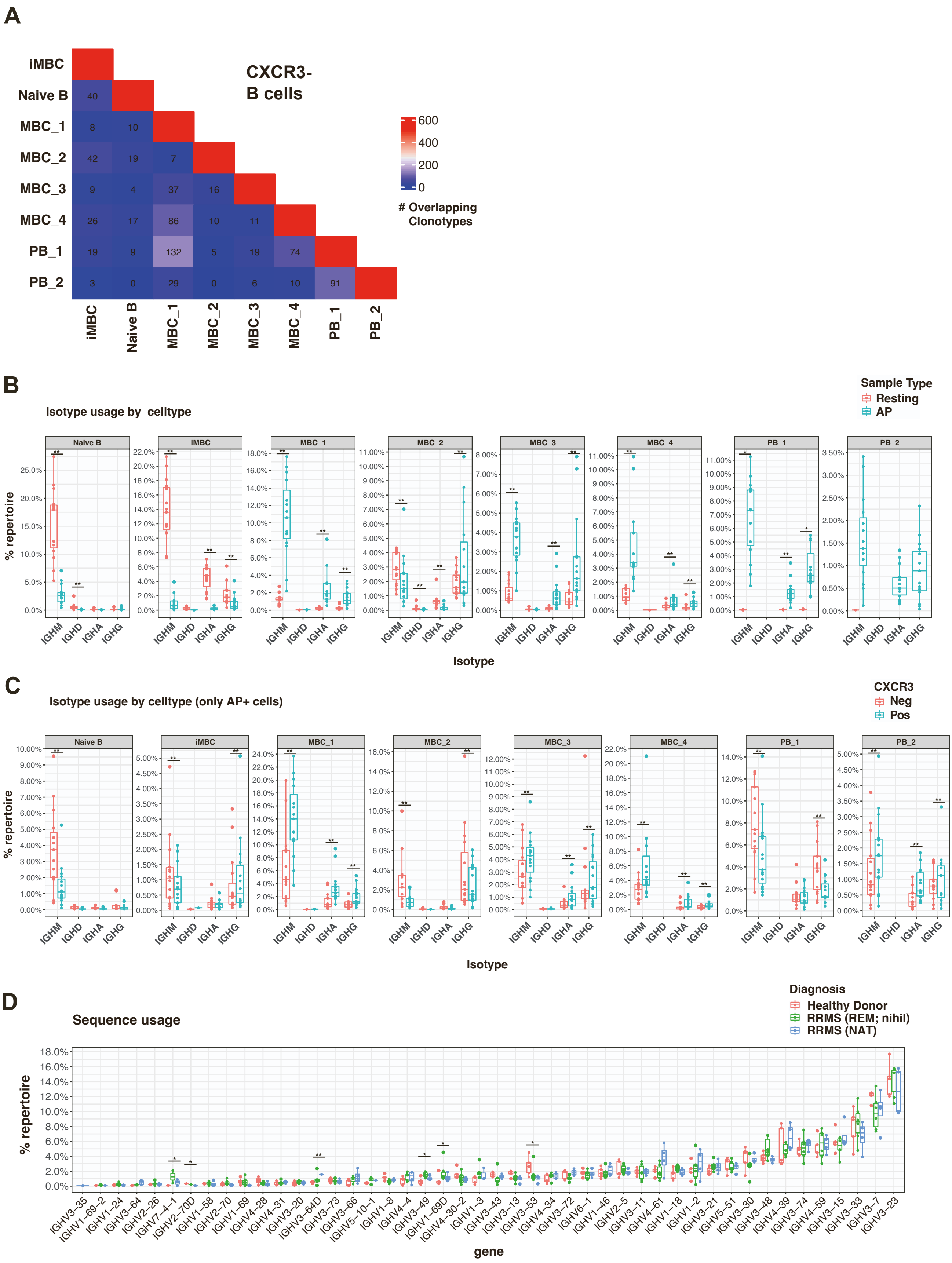

A
CXCR3-
B cells
iMBC
Naive B
MBC_1
MBC_2
MBC_3
MBC_4
PB_1
PB_2
Overlapping
Clonotypes
B
Isotype usage by celltype
Sample Type
Resting
AP
% repertoire
Isotype
IGHM
IGHD
IGHA
IGHG
C
Isotype usage by celltype (only AP+ cells)
CXCR3
Neg
Pos
D
Sequence usage
Diagnosis
Healthy Donor
RRMS (REM; nihil)
RRMS (NAT)
gene

Figure S6. Isotype and VDJ gene usage of CXCR3+ B cell subsets. Related to Figure 6, Table S1 and Table S4.

(**A**) Clonotype overlap between the various B cell subsets gated on CXCR3- B cells (related to Figure 6C). The color coding shows the degree of overlap and the numbers indicate shared clonotypes between the B cell subsets.

(**B, C**) *IGHM*, *IGHD*, *IGHA* and *IGHG* isotype proportions within the different B cell subsets comparing; (B) resting and AP compartment and (C) CXCR3- or CXCR3+ AP+ B cells. The percentage indicates the frequency of B cells with the specified isotype. Data includes all HD and MS patients from single-cell V(D)J sequencing (see Methods), HD (n = 5), REM (n = 6) and NAT (n = 5). Statistically significant groups (Fisher's exact test; FDR < 0.01) are marked with asterisk (* $p < 0.01$; ** $p < 0.001$).

(**D**) IGHV gene usage in CXCR3+ B cells across HD (n = 5), REM (n = 6) and NAT (n = 5) groups. Each donor is represented by a single dot in the box plots. Statistically significant groups (FDR < 0.05; two-sample T-test) are marked with asterisk (* $p < 0.05$; ** $p < 0.005$).

FIGURE S7

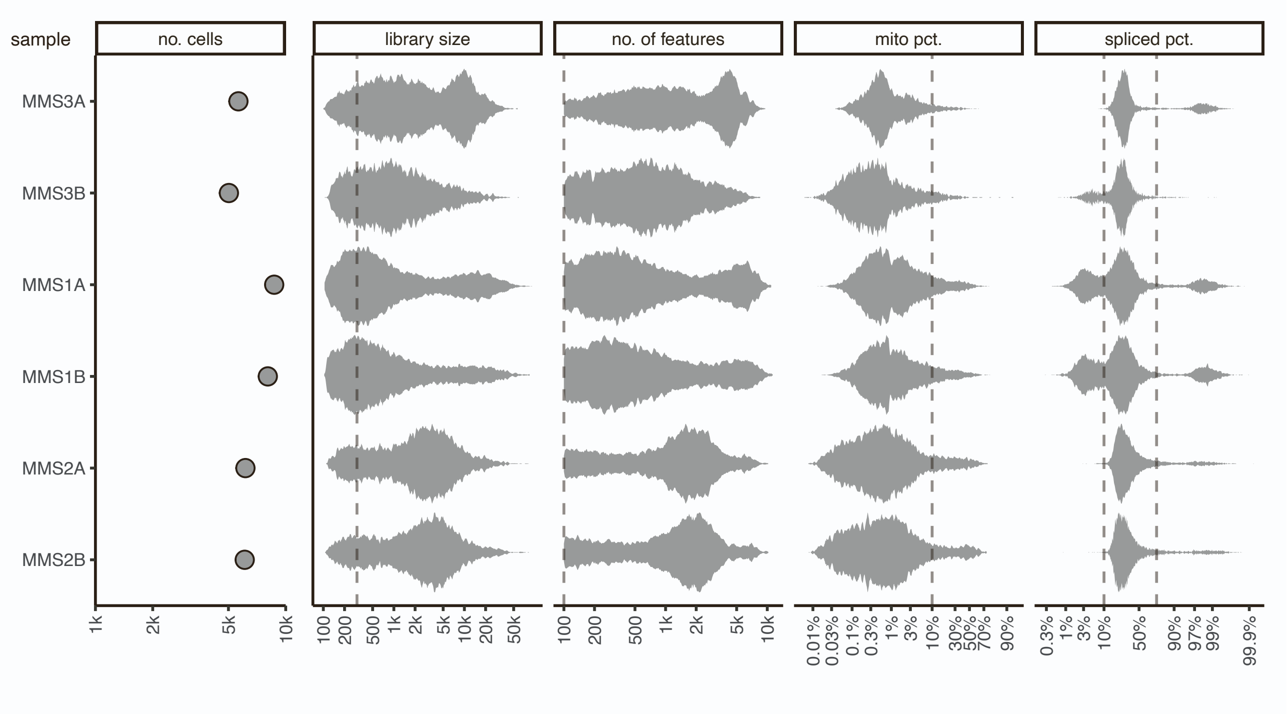
sample
no. cells
library size
no. of features
mito pct.
spliced pct.
MMS3A
MMS3B
MMS1A
MMS1B
MMS2A
MMS2B
1k
2k
5k
10k
100
200
500
1k
2k
5k
10k
20k
50k
100
200
500
1k
2k
5k
10k
0.01%
0.03%
0.1%
0.3%
1%
3%
10%
30%
50%
70%
90%
0.3%
1%
3%
10%
50%
90%
97%
99%
99.9%

Figure S7. Quantitative and qualitative assessment of single nuclei RNAseq. Related to Figure 7 and Table S5.

Nuclei from highly inflamed meningeal and adjacent grey matter tissue sections with well characterized high meningeal inflammation of 3 MS patients (MMS1: male, 40y, SPMS; MMS2: female, 44y, SPMS; MMS3: female, 53y, SPMS) were isolated twice (sample A and B) to run 2 biological replicate samples per donor based on the same brain tissue block. Around 5k nuclei were isolated for each sample, processed further for the generation of cDNA libraries, sequenced and subsequently analyzed for doublets, sample quality etc. prior to in depth analysis of the cluster annotation and expression data.

Table S1. Demographic characteristics of the study population, Related to all Figures and Tables.

Fig.	Donors	Treatment	n	age (mean±SD)	age (range)	F:M ratio	Treatment, duration (mean; range; in months)	HLA typing	*AP* *(Flow cytometry)*	4iTech	LEGEND screen	Soluble analytes (ELISA; LEGENDplex)	Functional assays (B-T Co-cultures; Inhibitor)	Genetic analysis (RNAseq; scRNAseq; Methylome)
1-5; S1-S4	HD	---	32	32.6 ± 6.8	25-49	1.7	none	X	X[a]			X[c]		
	RRMS (REL)	none/nihil[+]	18	33.1 ± 7.5	20-45	2.0	none	X	X[a]					
	RRMS (REM)	none/nihil[+]	35	37.7 ± 8.0	22-54	1.1	none	X	X[ab]		X	X[ce]	X[g]	X[h]
	RRMS	rituximab	14	47.3 ± 8.3	30-59	1.3	7.1; 3-21					X[c]		
	RRMS	natalizumab	15	36.6 ± 7.7	23-50	4.0	17.9; 11-30		X[a]	X		X[c]	X[fg]	
	PMS	none/nihil[+]	13	50.2 ± 9.0	26-65	0.9	none		X[a]					
4	HD	---	13	31.1 ± 4.8	25-41	0.9	none		X[a]			X[d]		
	RRMS (REM)	none/nihil[+]	16	34.3 ± 9.0	22-54	1.3	none		X[a]			X[de]		
	PMS	none/nihil[+]	5	54.8 ± 6.8	47-65	1.5	none		X[a]					
5	RRMS	natalizumab	6	28.0 ± 3.8	23-34	F only	28.3; 13-43		X (sort)					X[hi]
6; S5-S6	HD	---	5	32.4 ± 11.1	20-49	1.5	none	X	X (sort)					X[j]
	RRMS (REM)	none/nihil[+]	6	32.0 ± 9.0	22-48	2.0	none	X	X (sort)					X[j]
	RRMS	natalizumab	5	32.2 ± 7.6	23-42	1.5	15.8; 11-24	X	X (sort)					X[j]

HD = healthy donors; RRMS = relapsing remitting multiple sclerosis; PMS = progressive multiple sclerosis; REL = relapse; REM = remission; nihil = untreated;
F = female; M = male; [+]no glucocorticoids for at least 4 weeks and no other immunomodulatory treatments for at least 12 weeks; sort = Presorting of cell subpopulation performed
Flow cytometry using [a]surface marker labeling and [b]intracellular staining (incl. AID, pSTATs, T-bet, CXCL9); [c]CXCL9 ELISA; [d]Legendplex;
[e]CSF analytics (clinical liquor diagnostic) and clinical data; [f]B-T cell co-cultures, transfer experiments; [g]Inhibitor studies;
[h]RNAseq of pre-sorted B cells; [i]Methylome of pre-sorted B cells; [j]scRNAseq of pre-sorted B cells

Table S2. Demographic, clinical and radiological characteristics of MS patients with or without increased autoproliferation (AP). Related to Figure 2, Figure S2 and Table S1.
(**A**) Demographic data of patients with MS patients (RMMS and CIS) with low (<3.6% AP+ B cells) or increased (>3.6% AP+ B cells) B cell autoproliferation.
(**B**) Clinical and radiological parameters of RRMS and CIS patients at PBMC sampling (for AP assay).
(**C**) Clinical and radiological parameters of RRMS and CIS patients at last follow-up.

	**Patients with LOW AP+ B cells**	**Patients with HIGH AP+ B cells**	***p***
**A) RRMS/CIS patients**			
RRMS/CIS, n	17	17	n.a.
- RRMS, n (%)	16 (94.1%)	17 (100%)	n.a.
- CIS, n (%)	1 (5.9%)	0 (0%)	n.a.
Female/male ratio	1.4	1.4	n.a.
Age at disease onset (years), mean (±SD), median [±IQR]	32.1 (±7.5) 30.0 [±9.0]	29.4 (±9.5) 26.0 [±16.0]	0.1902*
**B) RRMS/CIS patients - Parameters at sampling**			
Age at sampling (years), mean (±SD), median [±IQR]	37.2 (±9.0) 37.0 [±11.0]	33.6 (±8.9) 31.0 [±11.0]	0.2077*
Disease duration (years) until sampling, mean (±SD), median [±IQR]	4.7 (±5.6) 2.0 [±8.0]	3.8 (±4.8) 1.0 [±8.0]	0.5687*
EDSS at sampling, mean (±SD), median [±IQR]	1.6 (±1.2) 1.5 [±1.5]	1.4 (±1.7) 1.0 [±2.0]	0.3628*
Global ARMSS (score) at sampling, mean (±SD), median [±IQR]	3.3 (±2.0) 2.9 [±3.1]	3.2 (±2.7) 2.4 [±5.0]	0.7279*
Updated MSSS (score) at sampling, mean (±SD), median [±IQR]	2.4 (±2.3) 2.6 [±3.9]	2.2 (±2.8) 0.9 [±4.3]	0.6527*
Patients with optic neuritis ever before sampling, n (%)	6 (35.3%)	6 (35.3%)	1.0000**
Patients with spinal syndrome ever before sampling, n (%)	8 (47.1%)	12 (70.6%)	0.2960**
Patients with spinal lesions on MRI around sampling, n (%)	10 (58.8%)	15 (88.2%)	0.1175**
Number of spinal lesions around sampling, mean (±SD), median [±IQR]	1.8 (±2.1) 1.0 [±3.0]	5.1 (±5.7) 3.0 [±6.0]	**0.0285***
**C) RRMS/CIS patients – Parameters at last follow-up**			
Age at last follow-up (years), mean (±SD), median [±IQR]	41.1 (±8.5) 42.0 [±13.0]	37.8 (±9.4) 36.0 [±8.0]	0.1802*
Disease duration (years) until last follow-up, mean (±SD), median [±IQR]	8.4 (±6.5) 8.0 [±7.0]	7.9 (±5.5) 6.0 [±8.0]	0.8103*

EDSS at last follow-up, mean (±SD), median [±IQR]	1.1 (±1.3)<br>1.0 [±1.5]	1.7 (±1.6)<br>1.5 [±1.0]	0.2041*
Global ARMSS (score) at last follow-up, mean (±SD), median [±IQR]	2.0 (±1.6)<br>2.3 [±2.1]	3.4 (±2.6)<br>2.9 [±5.1]	0.1260*
Updated MSSS (score) at last follow-up, mean (±SD), median [±IQR]	1.8 (±1.5)<br>1.4 [±2.0]	2.7 (±2.4)<br>2.1 [±3.9]	0.3628*
Patients with progression occurring until last follow-up, n (%)	1 (5.9%)	2 (12%)	1.0000**
Number of relapses since first relapse, mean (±SD), median [±IQR]	2.8 (±3.3)<br>2.0 [±2.0]	2.5 (±2.4)<br>2.0 [±3.0]	1.0000*
Patients with optic neuritis ever before last follow-up, n (%)	6 (35.3%)	5 (29.4%)	1.0000**
Patients with spinal syndrome ever before last follow-up, n (%)	10 (58.8%)	11 (64.7%)	1.0000**
Patients with spinal lesions on MRI around last follow-up, n (%)	12 (70.6%)	15 (88.2%)	0.3983**
Number of spinal lesions around last follow-up, mean (±SD), median [±IQR]	2.1 (±2.2)<br>1.0 [±6.0]	6.5 (±7.3)<br>6.0 [±6.0]	**0.0160***
Patients with brain stem syndrome ever before last follow-up, n (%)	7 (41.2%)	4 (23.5%)	0.4646*
Patients with brain stem lesions on MRI around last follow-up, n (%)	9 (52.9%)	12 (70.6%)	0.4813*
Number of brain stem lesions around last follow-up, mean (±SD), median [±IQR]	1.5 (±2.0)<br>1.0 [±2.0]	2.4 (±2.4)<br>2.0 [±5.0]	0.2340*

ARMSS – age-related MS severity scale according to Manouchehrinia et al., 2017
EDSS – expanded disability status scale
IQR – interquartile range
MSSS – multiple sclerosis severity score according to Roxburgh et al., 2005
SD – standard deviation
* Mann Whitney U test
** Fisher's exact test
